# A Biochemical Genomics Screen for Substrates of Ste20p Kinase Enables the *In Silico* Prediction of Novel Substrates

**DOI:** 10.1371/journal.pone.0008279

**Published:** 2009-12-16

**Authors:** Robert B. Annan, Anna Y. Lee, Ian D. Reid, Azin Sayad, Malcolm Whiteway, Michael Hallett, David Y. Thomas

**Affiliations:** 1 Department of Biochemistry, McGill University, Montreal, Quebec, Canada; 2 McGill Centre for Bioinformatics, McGill University, Montreal, Quebec, Canada; 3 Biotechnology Research Institute, National Research Council, Montreal, Quebec, Canada; 4 School of Computer Science, McGill University, Montreal, Quebec, Canada; 5 Rosalind and Morris Goodman Cancer Centre, McGill University, Montreal, Quebec, Canada; Leeds Institute of Molecular Medicine, United Kingdom

## Abstract

The Ste20/PAK family is involved in many cellular processes, including the regulation of actin-based cytoskeletal dynamics and the activation of MAPK signaling pathways. Despite its numerous roles, few of its substrates have been identified. To better characterize the roles of the yeast Ste20p kinase, we developed an *in vitro* biochemical genomics screen to identify its substrates. When applied to 539 purified yeast proteins, the screen reported 14 targets of Ste20p phosphorylation. We used the data resulting from our screen to build an *in silico* predictor to identify Ste20p substrates on a proteome-wide basis. Since kinase-substrate specificity is often mediated by additional binding events at sites distal to the phosphorylation site, the predictor uses the presence/absence of multiple sequence motifs to evaluate potential substrates. Statistical validation estimates a threefold improvement in substrate recovery over random predictions, despite the lack of a single dominant motif that can characterize Ste20p phosphorylation. The set of predicted substrates significantly overrepresents elements of the genetic and physical interaction networks surrounding Ste20p, suggesting that some of the predicted substrates are *in vivo* targets. We validated this combined experimental and computational approach for identifying kinase substrates by confirming the *in vitro* phosphorylation of polarisome components Bni1p and Bud6p, thus suggesting a mechanism by which Ste20p effects polarized growth.

## Introduction

Protein phosphorylation is a central post-translational modification in signal transduction; underscoring its importance is the observation that roughly 2% of eukaryotic genes encode kinases, and roughly one-third of all intracellular proteins may be phosphorylated on at least one residue [1]–[3]. However, given the large number of possible substrates for each of the many protein kinases, it is not surprising that the identification of kinase-substrate relationships remains a daunting challenge.

Our knowledge of kinase-substrate relationships has expanded using approaches that detect *in vitro* phosphorylation or *in vivo* phosphoproteins. *In vitro* methods include the use of purified kinases and substrates, kinases engineered to use ATP analogues, or phage display libraries [4]–[7]; peptide microarrays have been used to perform *in vitro* screens for kinase substrates in a high throughput manner [8], [9]. *In vivo* methods include the use of mass spectrometry to generate large-scale profiles of cellular phosphoproteins (reviewed in [10], [11]). Recent studies have combined these approaches with the examination of evolutionary conservation and interaction networks to better understand kinase-substrate relationships [12].While such approaches have expanded our knowledge of kinase-substrate relationships, it is clear that many remain unidentified or uncharacterized by current methods.

Bioinformatics techniques are increasingly employed to help identify kinase-substrate relationships, usually by characterizing the phosphorylation motif (i.e. describing the sequence at the site of phosphorylation) associated with a given kinase. For example, the amino acid preferences at the phosphorylation sites of a given kinase can be determined with a peptide library screen (e.g. [13]) and encoded in a position-specific scoring matrix (PSSM). Alternatively, it may be possible to characterize the phosphorylation motif with a regular expression (e.g. [ST]P.[RK] for CDK). Thus, any protein can be assessed for the likelihood that it can be phosphorylated by a specific kinase, based on whether its sequence possesses a likely phosphorylation site of the kinase. Since phosphorylation motifs are often highly degenerate, approaches based on these motifs rely on sophisticated machine learning techniques to increase the accuracy of substrate prediction; nevertheless, these approaches have proven most effective for the few kinases with the least degenerate motifs [14]–[18]. Recent approaches take into consideration repeat occurrences of a phosphorylation motif within a protein sequence. Modeling the propensity for such clusters of phosphorylation motifs results in improved accuracy for the prediction of CDK substrates, for instance [19], [20]. These studies raise the question of whether considering the co-occurrence of different motifs will also result in more accurate prediction of substrates for kinases with degenerate phosphorylation motifs.

Even in cases where phosphorylation motifs can be readily identified in putative substrates, the motifs do not generally provide sufficient information to unambiguously identify the physiologically relevant kinase. It has been recognized that sequence features that are independent of the actual phosphorylation site are often crucial for the phosphorylation of a substrate, including features that enable binding of the substrate to the regulatory domain of the kinase, binding of kinase and substrate to the same scaffold protein, or co-localization in the cell of kinase and substrate due to independent interactions (reviewed in [21]–[25]). Moreover, it has been recognized that kinases often bind substrates at a second site, distal to the active site, and that these docking interactions are largely responsible for kinase-substrate specificity [26]. These findings suggest that a predictor that takes into consideration such distal motifs, in addition to putative phosphorylation motifs, could produce more accurate predictions of kinase-substrate relationships.

The *Saccharomyces cerevisiae* Ste20p kinase (SGDID∶S000000999) is the founding member and prototype of the Ste20/PAK family, a large family of kinases ubiquitous in the genomes of all eukaryotes (for reviews see [27], [28]). Ste20p was first described as an activator of the yeast pheromone response MAPK cascade, and was subsequently also shown to activate the MAPK cascades responsible for pseudohyphal growth and the high-osmolarity glycerol (HOG) response [29]–[33]. Ste20p also regulates other physiological processes, such as actin cytoskeleton organization and polarized morphogenesis [34]–[36], mitotic exit [37], and hydrogen-peroxide induced apoptosis [38]. Furthermore, Ste20p shares an undefined essential role with its homolog Cla4p (SGDID∶S000005242), as *ste20Δ cla4Δ* mutants are not viable [39]. Despite the breadth of knowledge about the cellular roles of Ste20p, only a few of its substrates have been identified. In addition to its phosphorylation of Ste11p (SGDID∶S000004354) in the activation of MAPK pathways [33], Ste20p phosphorylates type I myosins Myo3p (SGDID∶S000001612) and Myo5p (SGDID∶S000004715) to promote actin polarization [40], [41], Cdc10p (SGDID∶S000000595), albeit less efficiently than Cla4p [42], and the histone H2B (SGDID∶ S000000098) [38]. Given the still-unidentified essential function it shares with Cla4p and the number of cellular processes in which it participates, it is likely that physiologically relevant substrates of Ste20p remain to be identified.

Our goal was to develop a method to facilitate the discovery of kinase-substrate relationships. Taken together, existing studies suggest that an *in silico* approach based on sequence motifs, characterizing phosphorylation sites and distal sites, may be useful for predicting the substrates of kinases that have not been well-characterized by existing methods. We used such an approach to identify substrates of the yeast Ste20p kinase.

## Results

### A Biochemical Genomics Screen Identifies *In Vitro* Substrates of the Ste20p Kinase

To develop an *in silico* tool for identifying substrates of the yeast Ste20p kinase, our first step was to generate a learning set of positive and negative examples of substrates. We designed a biochemical genomics screen to identify the *in vitro* substrates of kinases. This approach was applied to 539 yeast proteins coded by essential genes to identify substrates of Ste20p (see Materials and Methods). Essential genes were chosen to identify potential substrates responsible for the shared essential function of *STE20* and its homologue *CLA4* [39]. Individual clones expressing GST-fusion constructs under the control of the inducible *GAL1* promoter [9] of each of the designated proteins were grown under non-inducing conditions until mid-log phase. These were then combined in pools of eight, induced for three hours by the addition of galactose, and immobilized on glutathione-Sepharose beads. The combined pools of purified proteins bound to beads were incubated in each of two solutions: a solution containing the kinase domain of Ste20p, expressed and purified from *E. coli*, with necessary cofactors and γ-[P^32^]-ATP, and a control solution lacking Ste20p kinase. After thirty minutes, the samples were boiled in sample loading buffer and separated by SDS-PAGE, with subsequent visualization of phosphorylation by autoradiography. Where phosphorylation was observed, stepwise deconvolution confirmed the phosphorylation and identified the phosphorylated proteins.

Among the 539 proteins screened, 14 (2.6%) were reproducible *in vitro* substrates of Ste20p (Table 1). These were subsequently screened *in vitro* with Cla4p; as shown in Table 1, 10 of the 14 Ste20p substrates were also phosphorylated by Cla4p. Since Ste20p and Cla4p are known to share an uncharacterized essential function in yeast, suggesting that they share common targets, it is not surprising that several substrates are phosphorylated by both kinases. Nonetheless, Ste20p also exhibits specificity, as four of the 14 Ste20p substrates were not phosphorylated by Cla4p.

**Table 1:** Hits from the *in vitro* screen for Ste20p substrates.

Gene	Ste20p*	Cla4p*	Function/Process	PredictorScore
*ALY2*	Y	Y	interacts with CDK Pcl7p, unknown function	1.00
*BMS1*	Y	N	GTPase involved in ribosome biogenesis	1.00
*CDC3*	Y	Y	Septin	1.00
*COG4*	Y	Y	member of the Golgi complex involved in transport	0.99
*PEM1*	Y	N	phosphoacetyl-glucosamine mutase	0.99
*RAD53*	Y	Y	DNA damage checkpoint kinase	1.00
*RPT5*	Y	Y	26S proteasome regulatory subunit	1.00
*RSC6*	Y	N	component of the RSC chromatin remodeling complex	0.28
*RSC8*	Y	Y	component of the RSC chromatin remodeling complex	0.03
*SGV1*	Y	Y	nuclear cyclin-dependent kinase	1.00
*SPB1*	Y	Y	methyltransferase involved in ribosome biogenesis	1.00
*SPT16*	Y	Y	component of FACT complex involved in transcription elongation	0.03
*UTP5*	Y	Y	member of the SSU processome involved in ribosome biogenesis	0.03
*UTP7*	Y	N	member of the SSU processome involved in ribosome biogenesis	1.00

* Indicates whether the gene product is phosphorylated by the kinase of the column.

### An *In Silico* Approach to the Identification of Ste20p Substrates

Our goal was to build a Ste20p substrate predictor that considers sequence features of phosphorylation site and distal sites. In a naïve Bayes classifier, we thus integrated previously-defined PSSMs characterizing the phosphorylation sites of human Ste20p-related kinases [13] with motifs that characterize the substrates of Ste20p identified in our biochemical screen. For the latter component, we identified short sequence motifs, characterized with regular expressions, that are enriched in the set of Ste20p *in vitro* substrates (i.e. the positive learning set) relative to the screened set of proteins that were found not to be substrates (i.e. the negative learning set). Five substrates from the literature that had not been included in our initial screen (Htb2p, Myo5p, Myo3p, Ste11p, and Cdc10p [33], [38], [40], [42]) were added to the positive learning set. We enumerated the motifs that occur in multiple members of the positive learning set (via the pattern identification algorithm Teiresias [43]), specifically within regions of the protein sequences that were predicted to be exposed (according to ACCpro 4.0 [44]), and these motifs were evaluated for inclusion in a Ste20p substrate predictor.

**Figure 1:**
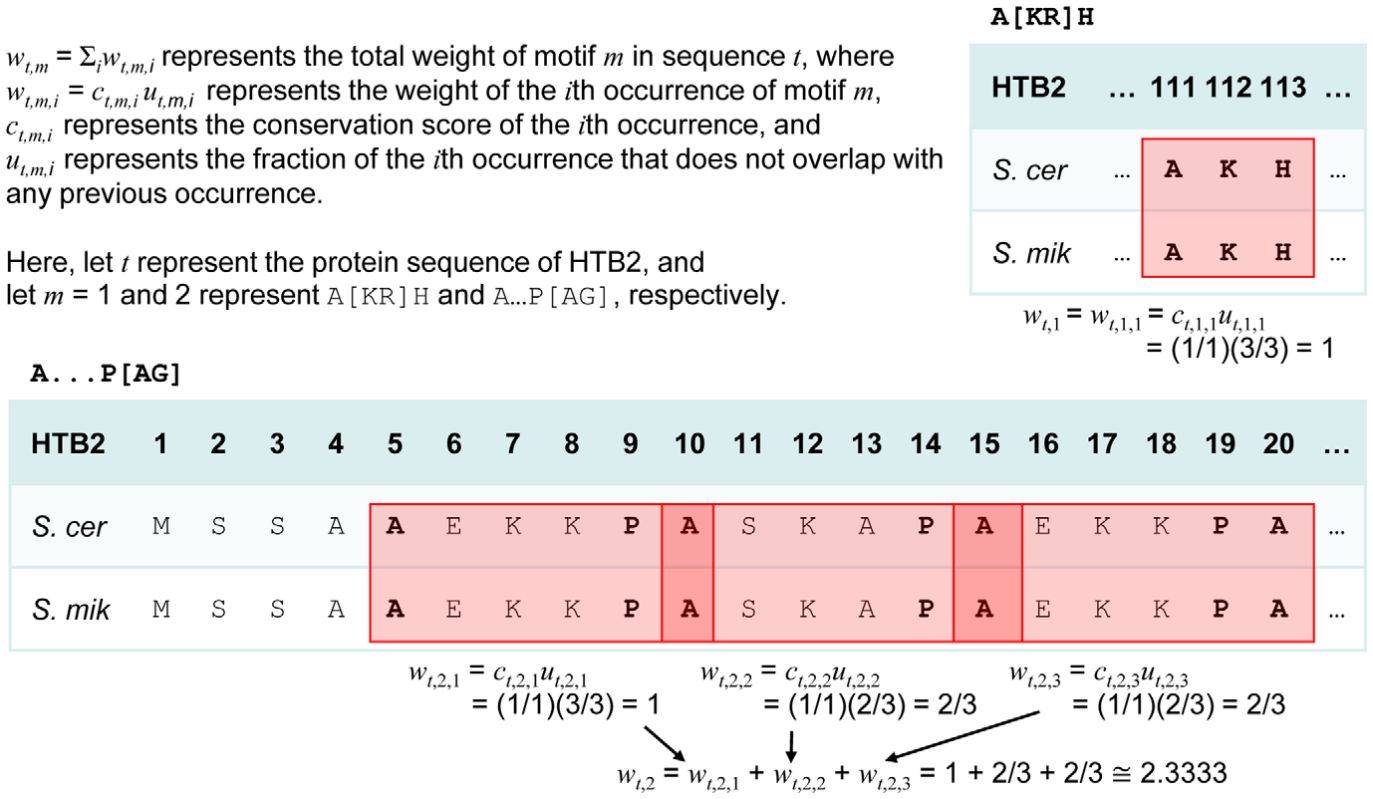
Examples for computing the total weight of a motif *m* for a given protein sequence *t* (*w_t,m_*). The sequence for Htb2p has occurrences of two different motifs used by the predictor: A…P[AG] and A[KR]H. There are three occurrences of the first motif in the sequence, and these occurrences overlap. The weight incorporates the conservation of the motif occurrences in other *Saccharomyces* species. Htb2p has an identified ortholog in only one other species in this genus: *S. mikatae*. Abbreviations: *S. cer* = *S. cerevisiae; S. mik* = *S. mikatae*.

Functionally important motifs are expected to be evolutionarily conserved. To bias our approach towards motifs that appear functionally important, we assigned each motif occurrence a weight ranging from 0 to 1 reflecting the degree of conservation across *Saccharomyces* species (*w_t,m,i_* in Figure 1; see Materials and Methods). The total weight of a motif for a given protein (*w_t,m_* = Σ*_i_w_t,m,i_* in Figure 1) is thus an estimate of the number of functionally important motif occurrences in the protein sequence. For each motif, we used its corresponding weights across the positive and negative learning sets to calculate a selectivity ratio that measures how frequently the motif occurs in the positive learning set as compared to how frequently it occurs the negative learning set (see Materials and Methods). As such, a motif with a selectivity ratio greater than one indicates that the motif is more prevalent in the positive set than in the negative set.

The 23 motifs with a selectivity ratio of at least 10 (Table 2) were integrated into the predictor such that occurrences of any of the motifs within a given amino acid sequence contribute to the belief that the sequence encodes a Ste20p substrate. Essentially, the predictor takes any peptide/protein sequence and returns the posterior probability that it represents a Ste20p substrate. We experimented with different parameter values to balance the trade-off between the sensitivity and specificity of the predictor (see Materials and Methods).

**Table 2:** Motifs used in the construction of the Ste20p substrate predictor.

Motif	Frequency in Positive Set*	Frequency in Negative Set*	Selectivity Ratio
K.H.V	0.2421	0.0141	17.18
KG..R	0.3596	0.0217	16.56
H[AG]..R	0.2158	0.0136	15.92
[ST]V.H	0.2982	0.0192	15.55
A...PG	0.3842	0.0282	13.64
AQR	0.4211	0.0310	13.58
[KR]...HR	0.2711	0.0208	13.04
K..HS	0.3298	0.0261	12.65
N.[KR]..H	0.3956	0.0322	12.29
P.G.Q	0.2149	0.0181	11.86
Q.DP	0.3289	0.0277	11.86
A..PP	0.2939	0.0248	11.85
E.C..[KR]	0.2237	0.0199	11.22
PG...S	0.3667	0.0332	11.05
G.NF	0.2404	0.0218	11.02
PT..Y	0.2982	0.0275	10.86
I..T.H	0.1711	0.0158	10.84
R.S..H	0.2807	0.0261	10.76
A[KR]H	0.2263	0.0212	10.67
G.K.P	0.2035	0.0197	10.32
A...P[AG]	0.5860	0.0569	10.29
K[AG]..R	0.5877	0.0572	10.27
RDA	0.2895	0.0283	10.23

* The frequency of motif occurrences in the positive/negative learning set, where each occurrence is weighted by its conservation across *Saccharomyces* species.

The accuracy of the predictor was tested *in silico* via a modified version of leave-one-out cross-validation (see Materials and Methods). The predictor is approximately as accurate as a variant of the predictor that only uses the 23 motifs identified in this study (see Figure S1 and Text S1). We thus used the variant as our definitive Ste20 substrate predictor in subsequent analyses for simplicity. The predictor has an estimated false positive rate of 11% and false negative rate of 74% if a protein is predicted as a substrate with a posterior probability of at least 0.9 (Figure S1). Moreover, the frequency with which we expect to identify true substrates within a set of predictions is 8% (i.e. the positive predictive value). This is roughly a three-fold enrichment over the frequency of experimentally verified Ste20p substrates among the initial selection of proteins in this study, and a five-fold enrichment over the frequency observed for an *in vitro* screen from a previous study [9].

### Application of the Predictor to the Yeast Proteome

We applied the predictor to the yeast proteome (6,696 proteins considered) and each yeast protein was ascribed a posterior probability that it is an *in vitro* substrate of Ste20p (Table S1). In total, 753 proteins (11.3%) were assigned a probability greater than 0.9, and 5050 proteins (75.4%) below 0.05. Amongst the 14 substrates identified in the initial biochemical genomics screen, ten scored with probabilities above 0.9 and three scored below 0.05 (Table 1). Previously described Ste20p substrates Ste11p, Myo3p, and Myo5p were all assigned a probability of 1.0, Cdc10 was assigned a probability of 0.86, and the histone Htb2p was assigned a probability of 0.035.

**Figure 2:**
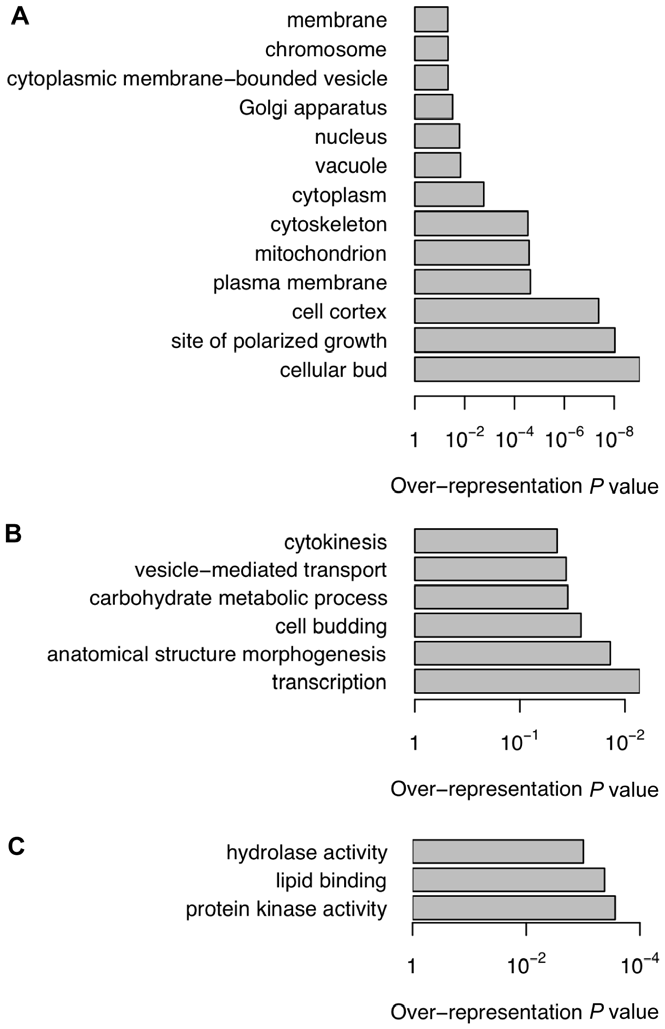
Significantly over-represented (adjusted *P*≤0.05) GO slim annotations among the predicted Ste20p substrates. (A) Cellular components. (B) Biological processes. (C) Molecular functions.

We performed pathway analysis on the predicted Ste20p substrates using the Gene Ontology (GO) [45]. The significantly overrepresented categories (adjusted *P *≤0.05) amongst the predicted substrates (score ≥0.9) are shown in Figure 2, Table S2, Table S3 and Table S4. Encouragingly, the cellular components and biological processes that are overrepresented overlap with the established role of Ste20p in budding and morphogenesis at sites of polarized growth, including the bud tip. Furthermore, the role of Ste20p as a component of several signaling cascades is reflected in the overrepresentation of proteins related to protein kinase activity amongst the predicted substrates. Thus, the biological relevance of our predictor is supported by the presence of a significantly high number of predicted substrates in process/pathways related to established roles of Ste20p.

### Genetic and Physical Networks Suggest *In Vivo* Relevance of Predicted Substrates

**Figure 3:**
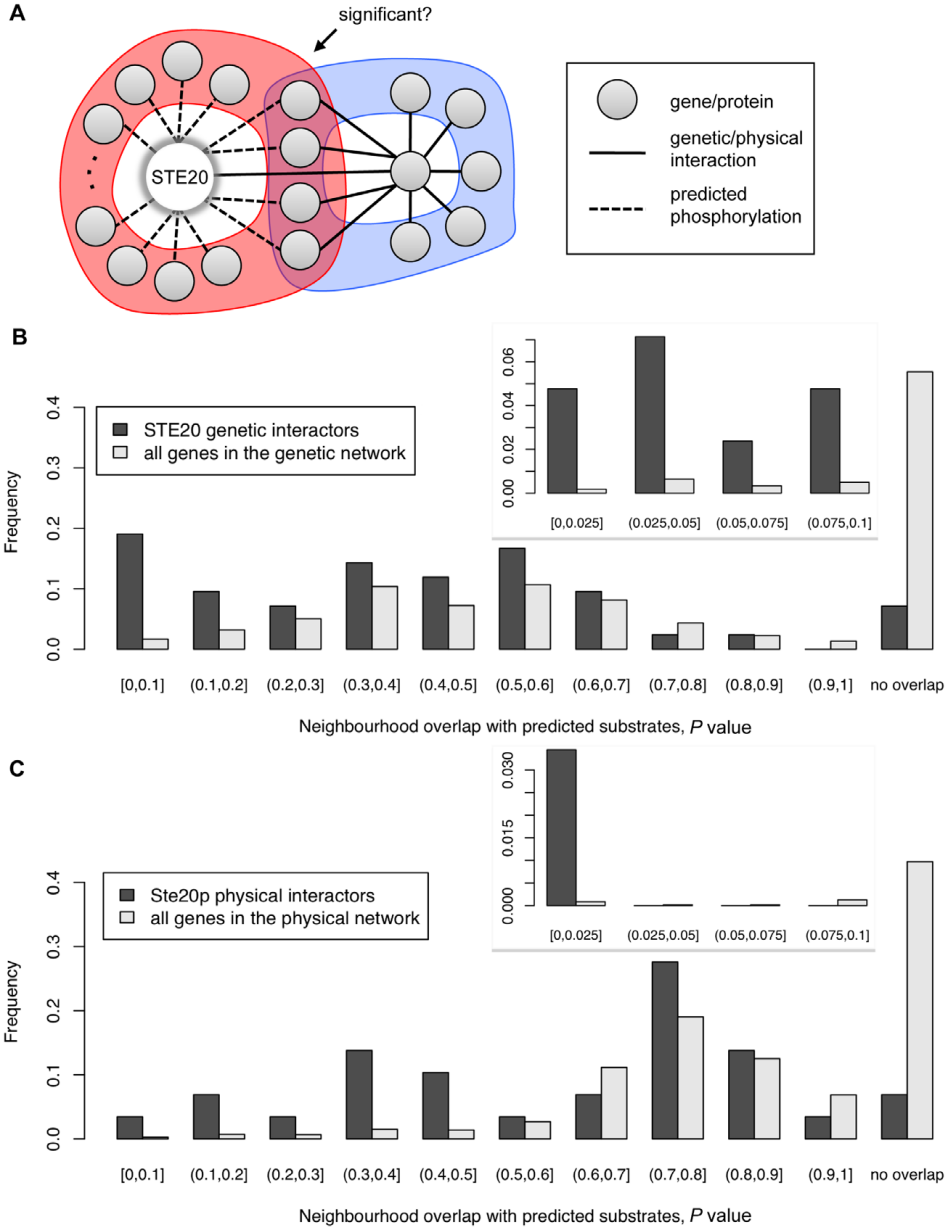
Inferring the biological relevance of Ste20p predicted substrates via neighborhood analysis. (A) Depiction of the test for the statistical significance of the overlap between the set of predicted substrates and the interaction neighborhood (blue portion) of a given gene/protein. Here the given gene is known to interact with *STE20*. (B) Neighborhood analysis in the context of the genetic network. Comparing the distributions of adjusted *P* values shows that the predicted substrates tend to overlap more significantly with the neighborhoods of *STE20* interactors versus those of all genes in the network. (C) Neighborhood analysis in the context of the physical network. A similar trend is apparent here but the *P* values are less extreme for Ste20p physical interactors. Insets for (B) and (C) depict the distributions at higher resolution where *P *≤0.1.

We reasoned that, since a kinase and a given substrate act in the same pathway, genes that genetically interact with *STE20* may also interact with the genes encoding *in vivo* substrates of Ste20p. Analogously, we also reasoned that proteins which form physical interactions with Ste20p binding partners (i.e. proteins in the neighborhoods of Ste20p physical interactors) are more likely to be accessible as substrates for Ste20p given that kinases and their cognate substrates often assemble in macromolecular complexes. To this end, we investigated whether any overlap exists between the network neighborhoods of *STE20* genetic and physical interactors and the set of predicted substrates (Figure 3A).

First, the predicted substrates were examined in the context of genetic interactions. A genetic interaction reflects a functional relationship between two genes determined by the level of some phenotype observed in the double mutant compared to the levels observed in the single mutants. We defined a network wherein each yeast gene is represented by a node and an edge is created between two genes if they are known to genetically interact (see Materials and Methods). For any node, the Genetic Interaction Neighborhood (GIN) is the set of nodes connected to it by an edge. The GIN of every gene in the network was tested for significant overlap with the set of predicted Ste20p substrates (Figure 3A). Indeed, the GINs of genes that genetically interact with *STE20* tend to overlap more significantly with the predicted substrates compared to the GINs of all other genes in the network (Figure 3B; *P *≈5.45×10^−4^, Mann-Whitney test, see Materials and Methods). Of the 42 published *STE20* genetic interactors, five of the corresponding GINs significantly overlap the set of predicted substrates (adjusted *P *≤0.05, hypergeometric test; Table S5). Given the expectation that *in vivo* substrates of Ste20p should share Ste20p's genetic interactions, the observed overlap between the predicted substrate set and the GINs of Ste20p's genetic interactors suggests that the *in silico* predictor exhibits *in vivo* relevance.

We next performed an analogous analysis based on physical interactions. Here we say a physical interaction exists between two proteins if they have been shown to physically interact directly or indirectly as part of the same complex. We thus defined a network where an edge was placed between two proteins if they are known to physically interact (see Materials and Methods). For any node, the Physical Interaction Neighborhood (PIN) is the set of nodes connected to it by an edge. The *Saccharomyces* Genome Database [46] contains 96 published physical interactors for Ste20p, although substrates identified in previous high-throughput studies (such as [8]) were excluded to avoid bias or redundancy. The physical interactors examined include, for example, the scaffold proteins for Ste20p-related signaling complexes such as Bem1p (SGDID∶S000000404) and Cdc24p (SGDID∶S000000039).

Although only one of the Ste20p PINs (i.e. the neighborhood of Cdc28p (SGDID∶S000000364)) significantly overlaps the set of predicted substrates (adjusted *P *≤0.05, Fisher's exact test; Table S6), the 29 PINs tend to have lower overlap *P* values than the PINs of the other proteins in the network (Figure 3C; *P *≈8.29×10^−4^, Mann-Whitney test, see Materials and Methods). In fact, as is the case with genetic interactions, the neighborhoods of the majority of proteins have no overlap with the set of predicted substrates, whereas 27 out of the 29 Ste20p PINs have some overlap (i.e. a significant number with *P *≈1.03×10^−4^, hypergeometric test). By combining the *in silico* predictor with GIN and PIN analyses, it becomes possible to focus on physiologically relevant potential substrates based on their additional biological connections to Ste20p.

**Figure 4:**
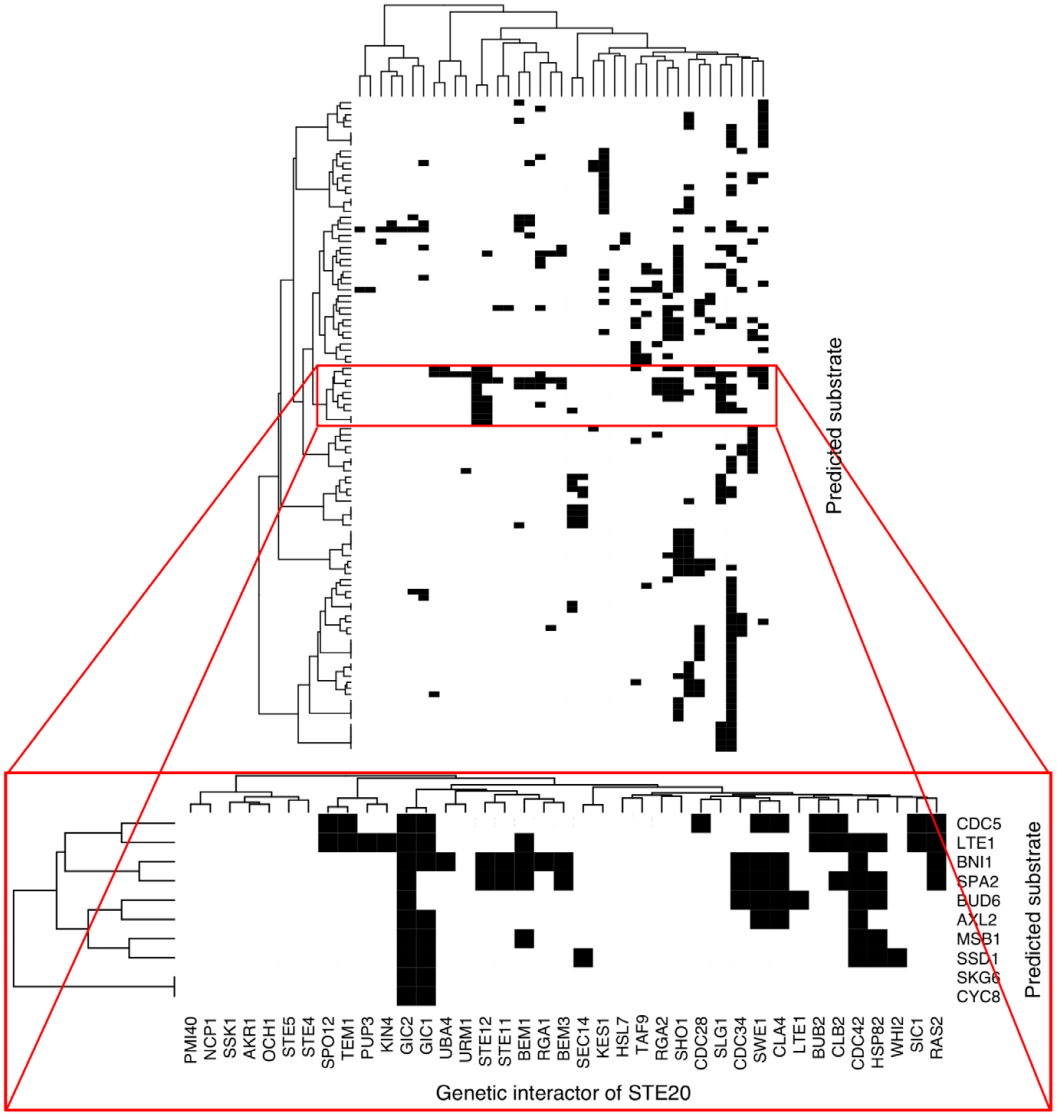
Clustering profiles of overlap between the predicted substrates and the GINs of *STE20* genetic interactors. A solid black cell indicates the presence of a predicted substrate (row) in a GIN (column). A cluster of predicted substrate profiles is shown at higher resolution. *SPA2* and *BNI1* form a significant subcluster (AU score = 97, Figure S2B) and they are involved with polarized growth.

**Figure 5:**
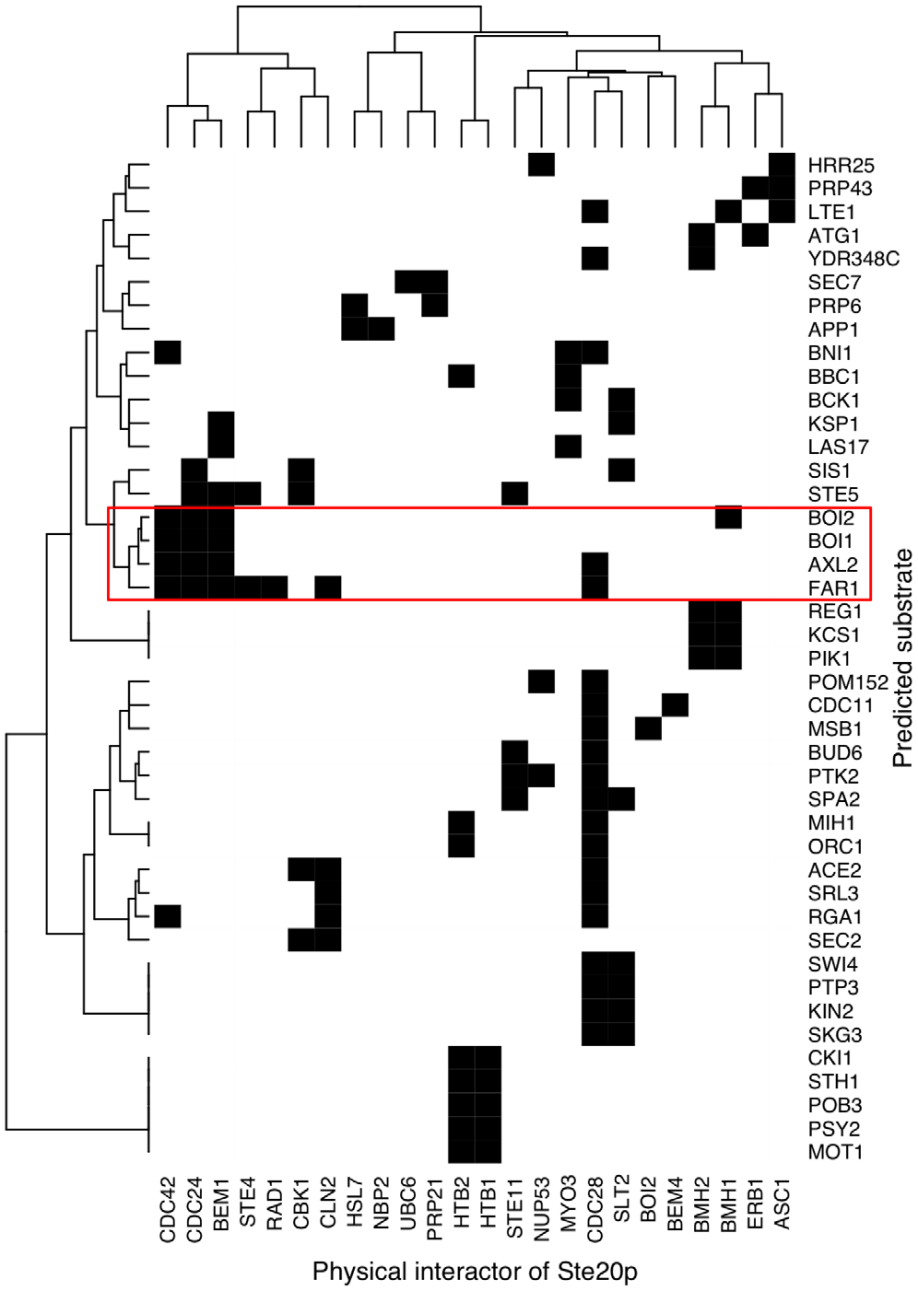
Clustering profiles of overlap between the predicted substrates and the PINs of Ste20p physical interactors. A solid black cell indicates the presence of a predicted substrate (row) in a PIN (column). Several predicted substrates implicated in polarized growth are clustered together (AU score = 94, Figure S3B). Highlighted in red is a subcluster of predicted substrates that are present in the PINs of Ste20p physical interactors that are also involved with polarity (Cdc42p, Cdc24p, Bem1p).

In order to better characterize the physiological relevance of the neighborhood approach, we clustered the genetic and physical interactors of *STE20* with respect to the predicted substrates in their neighborhoods (Figures 4 and 5, seeFigure S2 and S3 for the statistical significances of the clusters). In other words, *STE20* interactors with many common predicted substrates in their respective neighborhoods are likely to co-cluster. The clustering of the genetic interactors of *STE20* depicted in Figure 4 identifies one large cluster that includes genes coding for proteins involved in cell-cycle progression and polarized growth such as *CDC28*, *SWE1* (SGDID∶S000003723), *CLA4*, *CDC42* (SGDID∶S000004219), and *RAS2* (SGDID∶S000005042) [Approximately Unbiased (AU) score = 94 as shown in Figure S2A, see Materials and Methods]. Moreover, most of the genes in this cluster interact with a set of predicted substrates that also include polarity- and cell-cycle-associated genes such as *CDC5* (SGDID∶S000004603), *LTE1* (SGDID∶S000000022), *AXL2* (SGDID∶S000001402), and *MSB1* (SGDID∶S000005714) (highlighted in Figure 4). Also included in this list are three of the four known components of the polarisome (*BNI1* (SGDID∶S000005215), *SPA2* (SGDID∶S000003944), and *BUD6* (SGDID∶S000004311)), whose activation has been linked genetically to *STE20* [47].

This analysis of the Ste20p physical interactors also highlights a tight clustering of proteins related to polarized growth (Figure 5, Figure S3). Cdc42p, its guanine nucleotide exchange factor (GEF) Cdc24p, and the scaffold Bem1p are Ste20 interactors that co-cluster. These overlap with a significant cluster of predicted substrates (AU score = 94 as shown in Figure S3B) including the polarity proteins Boi1p (SGDID∶S00000018) and Boi2p (SGDID∶S000000916), suggesting that these may serve as physiologically relevant substrates of Ste20p. Polarisome components Bud6p and Spa2p cluster together with the kinase Ptk2p (SGDID∶S000003820), although Bni1p is clustered with another set of actin-associated proteins including Bbc1p (SGDID∶S000003557) and Las17p (SGDID∶S000005707). Thus, by combining the predicted biochemical relationships between Ste20p kinase and potential substrates with the known relationships of genetic and physical interactors of *STE20*, it becomes possible to identify novel roles for Ste20p phosphorylation *in vivo*.

### Polarisome Components Bud6p and Bni1p Are *In Vitro* Substrates of Ste20p

We sought to validate our approach to Ste20p substrate prediction by assaying several high-scoring proteins that are also present in *STE20* interactor neighborhoods. The neighborhood cluster analysis described above identified a number of putative substrates involved in polarized growth. Ste20p participates with Cdc42p in the establishment of polarized growth at directed sites in response to intrinsic budding cues and extrinsic signals such as mating pheromones or altered nutrient conditions [48]. In these processes, Ste20p has been genetically linked to the polarisome, a 12S macromolecular complex that controls polarized growth and morphogenesis [47], [49].

**Figure 6:**
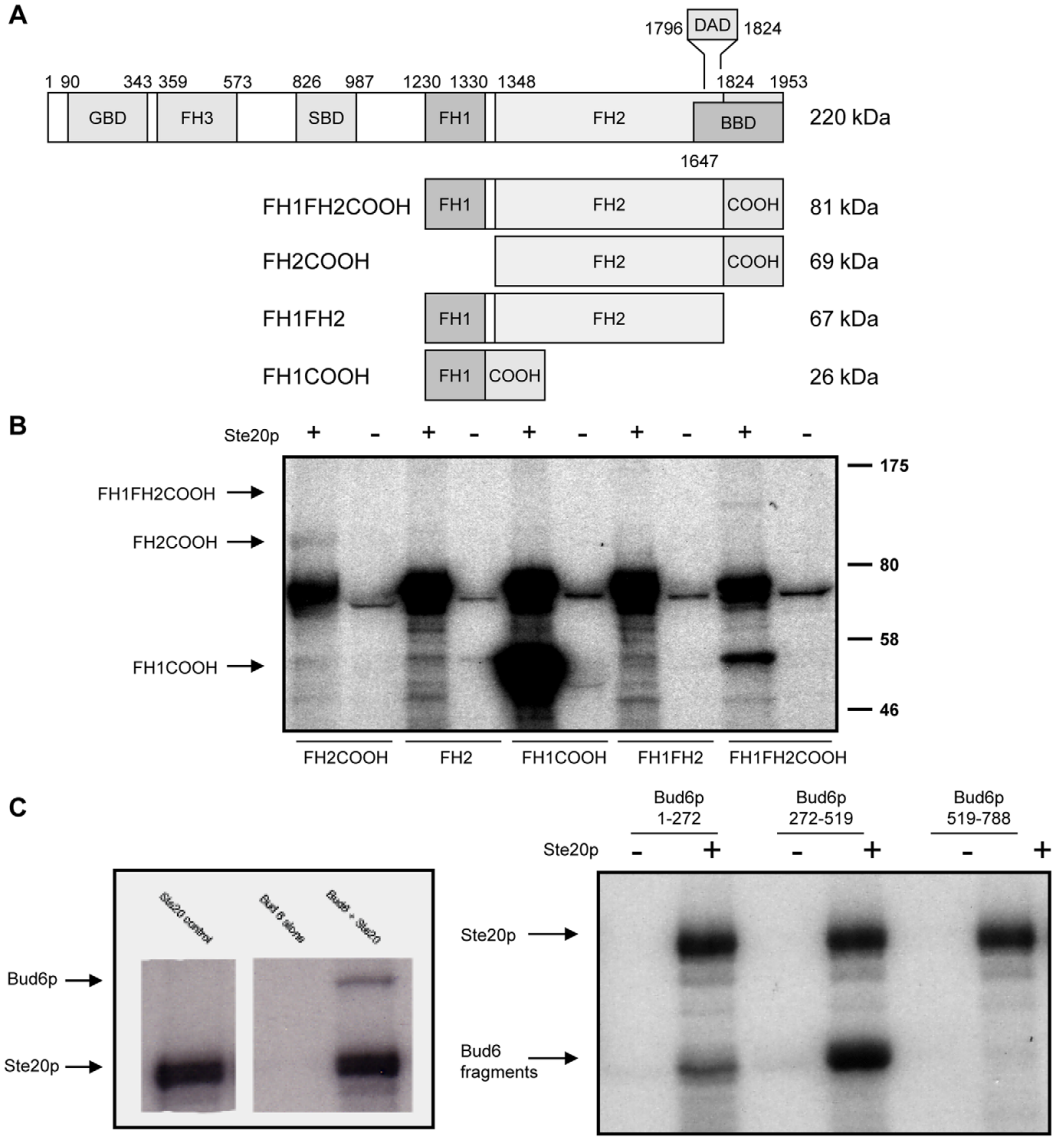
Bni1p and Bud6p are phosphorylated by Ste20p *in vitro*. (A) A schematic representation of the functional regions of Bni1p. These are the Formin-Homology domains (FH1, FH2, and FH3), GTPase binding domain (GBD), Spa2p-binding domain (SBD), Dia-autoregulatory domain (DAD), and Bud6p-binding domain. The region C-terminal to the FH2 domain, which contains part of the BBD, is referred to as the COOH region in the text (figure adapted from [66]). (B) Ste20p only phosphorylates Bni1p constructs containing the COOH region. Constructs composed of different combinations of the FH1 and FH2 domains and the COOH region were purified and equal concentrations of each were assayed in *in vitro* kinase assays with Ste20p and γ-[P32]-ATP, then visualized by SDS-PAGE and autoradiography. The three constructs containing the COOH regions are phosphorylated (with the position of the labeled peptides in their respective lanes indicated by arrows at left), whereas the constructs without the COOH region are not. (C) Ste20p phosphorylates the central region of Bud6p. In the left panel, full length Bud6p is phosphorylated by Ste20p. In the right panel, the middle fragment of Bud6p exhibits strong evidence of phosphorylation and the N-terminal fragment exhibits weak evidence of phosphorylation. No signal is detected for the C-terminal fragment.

Examination of the set of predicted Ste20p substrates revealed that three of the four polarisome components (Bni1p, Bud6p, and Spa2p) were predicted with high probability (>0.98) to be Ste20p substrates (Table S1). Furthermore, these three were also identified numerous times when cross-referenced against *STE20* interactor GINs and PINs; *BNI1* is a member of 16 neighborhoods, *SPA2* is a member of 15, and *BUD6* is a member of 10. We verified the predictions by performing *in vitro* kinase assays with the polarisome proteins to determine whether they could serve as substrates of Ste20p. The candidate substrates were exogenously expressed as GST-fusions, and assays were performed essentially as in the biochemical screen (see Materials and Methods). As shown in Figure 6, Bni1p and Bud6p are both phosphorylated by Ste20p *in vitro*. Spa2 was not phosphorylated by Ste20p (data not shown). Thus, both Bni1p and Bud6p were predicted and experimentally confirmed to be Ste20p substrates.

To gain greater insight into the consequences of Ste20p phosphorylation, we identified phosphorylation sites in Bni1p and Bud6p. Bni1p is a large protein (1,953 residues) which was previously shown to be a phosphoprotein *in vivo* whose phosphorylation is reduced in a *ste20Δ* mutant [47]. It comprises a N-terminal Cdc42p binding domain and three C-terminal regions characteristic of the formin-family of proteins, which together constitute the actin-assembly machinery. These C-terminal regions include the Formin-homology 1 and 2 (FH1 and FH2) domains, and a C-terminal tail region (COOH) that includes two domains: the Bud6-binding domain (BBD) and a *cis*-inhibitory Dia-autoregulatory domain (DAD) (Figure 6A). Given the functional importance of the C-terminal domains, we expressed subclones composed of the FH1, FH2, and COOH domains as GST-fusion proteins and repeated the *in vitro* Ste20p kinase assays. As seen in Figure 6B, phosphorylation was observed in the constructs containing the COOH region, but was not observed in constructs in which it is absent. Thus, Ste20p phosphorylation *in vitro* occurs within the region of Bni1p responsible for binding Bud6p.

Next, we sought to validate that Bud6p is phosphorylated *in vitro* by Ste20p (Figure 6C). While the domain organization of Bud6p is not as well-defined as for Bni1p, it has been determined that the C-terminal region (519–788aa) is involved in dimerization as well as binding Bni1p and actin, whereas the N-terminal region (1–166) is required for proper Bud6p localization [50]. We thus subcloned Bud6p to determine which regions are phosphorylated by Ste20p *in vitro*. As seen in Figure 6C, weak phosphorylation is observed in the N-terminal fragment, a stronger signal is observed in the uncharacterized middle region, and no phosphorylation is observed in the region involved in dimerization or binding to actin and Bni1p. Thus, while Ste20p phosphorylates Bni1p in the region responsible for Bud6p binding, it does not phosphorylate Bud6p in the region responsible for binding Bni1p.

We used mass spectrometry to identify *in vivo* phosphorylation sites for Bud6p. Using standard procedures (see [51] and Materials and Methods), we identified *in vivo* phosphorylation of a TAP-tagged Bud6p fusion protein on two residues: serine 327 and serine 342. These two residues are found in the middle fragment, which was phosphorylated by Ste20p *in vitro* (Figure 6C). Thus, Bud6p is a phosphoprotein *in vivo* and the phosphorylation on residues Ser327 and Ser342 correlates with the phosphorylation of the same region by Ste20p *in vitro*. Given that the region of Bni1p which is phosphorylated by Ste20p is required for viability in the absence of *CLA4* [47], we asked whether the same is true for Bud6p. Expression of a *bud6* construct with the region containing both phosphorylation sites deleted (*bud6^Δ272–411^*) retains the ability to rescue the lethality of a *bud6Δ cla4Δ* strain and results in a morphology similar to a *cla4Δ* mutant (data not shown). While the *in vivo* relevance of Bud6p phosphorylation remains to be determined, the substrate predictor correctly suggested that direct phosphorylation of polarisome proteins by Ste20p occurs *in vitro*, and thus presents opportunities for directed investigation of the activation mechanism of this complex.

## Discussion

We developed a strategy to aid the discovery of substrates for any given kinase and demonstrated its utility with the yeast Ste20p kinase. In particular, we tested ∼550 proteins in a biochemical screen for *in vitro* substrates of Ste20p. The results were used to generate an *in silico* predictor of Ste20p substrates. Cross-referencing the predicted substrates against known genetic and physical interactions highlighted polarisome components as likely targets of *in vivo* phosphorylation by Ste20p. Of these components, Bni1p and Bud6p were shown to be phosphorylated by Ste20p *in vitro*. The phosphorylation was mapped to a region of Bni1p which is genetically associated with *STE20*, and which binds to Bud6p. *In vivo* phosphorylation sites were identified on Bud6p, but the physiological relevance of these sites remains unclear.

The decision to screen proteins coded by essential genes was made to address the shared essential function of *STE20* and *CLA4* [39]. We reasoned that if the phosphorylation of a single substrate is responsible for the synthetic lethality of the *ste20Δ cla4Δ* mutant, then the gene coding for that substrate may also be essential. We thus screened roughly half the complement of yeast essential genes, selected on the basis of GO annotations that we assumed might overlap with known functions or localizations of Ste20p and Cla4p. As shown in Table 1, 10 of 14 substrates phosphorylated by Ste20p were also phosphorylated by Cla4p. It remains to be determined whether any of these substrates may be responsible for the shared essential function of Ste20p and Cla4p. While the identification and mutational analysis of *in vivo* phosphorylation sites on these substrates should yield insight into this question, our results also suggest the phosphorylation of Bni1p is related to this essential function.

Our approach to kinase-substrate identification can complement and support various other methods currently used to identify kinase substrates. For instance, an *in vitro* screening approach using protein microarrays [8] has identified 70 substrates for Ste20p, 11 of which were also tested in our screen. We confirmed a 36% recovery of chip-identified substrates in our screen, which is generally consistent with observed differences between related high-throughput assays and with the observation that different approaches to identify physical, genetic, and biochemical interactions are complementary to each other [52].

This earlier study also employed a pattern-searching algorithm to identify predictive phosphorylation motifs for each of the tested kinases. It succeeded in identifying phosphorylation motifs for 11 of the 87 kinases tested, suggesting these are kinases with strict phosphorylation site requirements [8]. Though they did not identify a Ste20p phosphorylation site motif, our multi-motif predictor of Ste20p substrates exhibits an estimated three-fold improvement, over screening randomly selected proteins, in the rate of substrate identification. This substrate enrichment is especially significant since the predictor is based on a screen of only 539 proteins. Thus, our approach improves upon previous efforts to capture sequence motifs that predict substrate status.

This improvement is likely due to the fact that our predictive approach does not rely on strict phosphorylation site requirements, but rather a set of sequence motifs that are not restricted to describing the phosphorylation site and can therefore predict substrates by other means. We reasoned that, while no individual sequence feature may be sufficient to identify Ste20p substrates, the combination of relevant motifs may allow for substrate prediction with a higher degree of accuracy.

Our approach identified 23 motifs that were then used in a naïve Bayes classifier to assign a posterior probability of being a Ste20p substrate to each member of the proteome. Amino acid preferences at the phosphorylation sites of human Ste20p-related kinases have been specified via position-specific scoring matrices (PSSMs) [13], and some of the motifs identified in this study capture the predominant preference for basic amino acids at positions N-terminal to the sites (i.e. K..HS and R.S..H). Moreover, we showed that integrating the PSSMs with our motifs does not improve the accuracy of our Ste20p substrate predictor. This suggests that our motifs sufficiently capture the preferences specified by the PSSMs that are useful for predicting Ste20p substrates. It remains to be determined how these motifs participate in specifying Ste20p phosphorylation, whether via *cis* effects through a docking interaction with Ste20p, or *trans* effects through binding with a Ste20p-associated scaffold such as Bem1p, Ste5p (SGDID∶S000002510), or Far1p (SGDID∶S000003693). Though the molecular functions of the motifs are not yet clear, our analyses showed significant associations between the set of predicted substrates and genes/proteins that are already (directly or indirectly) associated with *STE20*. Thus, although the predictor is based on the *in vitro* biochemistry and primary structure of proteins, employing multiple evolutionarily-conserved selective motifs seems to result in biologically relevant substrate predictions.

While the predictor remains a tool for identifying a biochemical relationship between a kinase and its potential substrates, providing biological context, for example by pathway analysis, suggests hypotheses that physiologically relate predicted substrates to kinases. While pathway analysis reveals that the predicted Ste20p substrate set appears consistent with known roles of Ste20p, the analysis also suggests a potential role for Ste20p in vesicle-mediated transport, which is supported by the observation that the human Ste20p-related kinase Pak1 plays a role in regulating vesicular-based transport in human fibroblasts [53]. Likewise, a role for Ste20p in carbohydrate metabolic processes is supported by the observation that Pak1 phosphorylates and activates phosphoglucomutase-1 (PGM; Ensembl∶ENSG00000079739) [54].

The biological or *in vivo* relevance of the predicted substrates was also evaluated using the genetic and physical interaction networks surrounding Ste20p. Combining genetic and physical interaction data with biochemical data has been shown to be an effective means of evaluating and assigning biological relevance to observed phosphorylation. For instance, the NetworKin methodology employs several types of data in order to assign thousands of identified *in vivo* phosphorylation sites to the roughly 500 human kinases [55]. In that framework, genetic and physical interactions are used to evaluate possible kinase-substrate relationships. Here, we employ a similar approach to evaluate substrates that have been predicted computationally. The analysis focused on genes/proteins that are closely linked to *STE20* in the genetic and physical interaction networks, reasoning that these are the most likely to represent strong candidates for biologically relevant associations with the kinase. Indeed, *STE20* interactor GINs tend to overlap more significantly with the predicted substrate set compared to the GINs of other genes in the network. Analogously, Ste20p interactor PINs tend to overlap more significantly than other PINs with the predicted substrate set. The interaction neighborhood analyses therefore support the hypothesis that the predicted substrates represent candidates for *in vivo* phosphorylation by Ste20p.

Large-scale screens and bioinformatics analyses are a great source of novel biological hypotheses, and our analyses led to the hypothesis that Ste20p phosphorylates components of the polarisome complex. Here, we confirmed that Bni1p and Bud6p are phosphorylated by Ste20p *in vitro*, but that the other two components are not. Spa2p was assigned a high posterior probability of being a Ste20p substrate and is also present in many Ste20p interactor neighborhoods. It may therefore represent a false prediction. However, it is also possible that there are additional requirements for Spa2p phosphorylation by Ste20p that are not present in the *in vitro* assay. Nonetheless, our predictive method resulted in the identification of two novel substrates for Ste20p phosphorylation.

The Ste20p phosphorylation of Bni1p occurs in the C-terminal Bud6-binding domain (BBD). It has been previously shown that expression of a *bni1* construct lacking this BBD region is unable to rescue the synthetic lethality of a *bni1Δ cla4Δ* mutant, and therefore the transformed mutants exhibit a terminal phenotype similar to that of *ste20Δ cla4Δ* mutants [47]. The phenotypic similarity between strains lacking the region of Bni1p phosphorylated by Ste20p and those lacking Ste20p altogether suggests that phosphorylation of this region occurs *in vivo* and is required in the absence of *cla4Δ*. As its name implies, this region also binds the other polarisome substrate of Ste20p, Bud6p, indicating the potential for sophisticated regulatory coordination. Though the *in vivo* relevance of Ste20p phosphorylation and the coordination of the phosphorylation of Bni1p and Bud6p in the regulation of polarized growth remain to be determined, our predictive approach to substrate identification has provided a framework for further investigation.

Phosphorylation is a key modification involved in signal transduction, and thus participates in many of the dynamic processes of the cell. Despite this importance, identifying the detailed architecture of phosphorylation networks remains a challenge. Here, we have described a combination of biochemical genomics and bioinformatics to identify potential new substrates for the Ste20p kinase in yeast. We have confirmed experimentally that our approach predicts valid *in vitro* Ste20p substrates, and leads to greater insight into the functions of this kinase.

## Materials and Methods

### Materials

Restriction endonucleases and DNA-modifying enzymes were obtained from New England Biolabs and GE Healthcare. Protease inhibitor tablets and reduced glutathione were obtained from Roche. Glutathione-Sepharose 4B beads were purchased from GE Healthcare. Radioisotopes were purchased from GE Healthcare and Perkin Elmer, and film for autoradiography was BioMax MS from Kodak. Acid-washed glass beads (450–600 µm), protease inhibitors, sorbitol, and trypsin were purchased from Sigma. The yeast GST-6xHIS Open Reading Frame collection was purchased from Open Biosystems.

### Construction of Plasmids

Yeast expression GST-fusion proteins were obtained from Open Biosystems [9]. The GST-Bni1 constructs were kindly provided by C. Boone (University of Toronto).

The Bud6p fragments were expressed in *E. coli*. Relevant fragments were amplified from genomic DNA [56]. pRA210 expresses full-length *BUD6* and was constructed using the oligonucleotides 5′-GAGACCCGGGGAATGAAGATGGCCGTGGATGACC-3′ and 5′-GAGACTCGAGTTAAGTAAACCCCGGCCCAAAATATGC-3′. pRA211 expresses *BUD6^1–272^* and was constructed using the oligonucleotides 5′-GAGACCCGGGGAATGAAGATGGCCGTGGATGACC-3′and 5′-GAGACTCGAGTTAAGCTTCTGTTGTAGACTGATTTGTC-3′. pRA212 expresses *BUD6^272–519^* and was constructed using the oligonucleotides 5′-GAGACCCGGGAGCTGCTGCGGCTGCCGGCCTCATGAC-3′ and 5′GAGACTCGAGTTACCTATTAATATTATGCACTTGTTT-3′. pRA213 expresses *BUD6^519–788^* and was constructed using the oligonucleotides 5′-GAGACCCGGGAAACAAGTGCATAATATTAATAGG-3′ and 5′-GAGACTCGAGTTAAGTAAACCCCGGCCCAAAATATGC-3′. The underlined nucleotides are *SmaI* or *XhoI* sites. The PCR products were inserted into the vector pGEX-5T by cutting both with *SmaI* and *XhoI* followed by ligation. The resultant plasmids were confirmed by sequencing.

### Yeast Strains and Protein Purifications

Yeast media, culture conditions, and manipulations were as described [57]. Transformation of yeast with plasmid DNA was achieved with lithium acetate and standard protocols [57].

Growth and induction of yeast strains for the biochemical screen were essentially as described [9]. Cell patches were inoculated in SD (2%) -ura medium, grown overnight, washed, reinoculated in raffinose (4%) -ura, and grown to an absorbance at 600 nm of 0.8. Cultures were pooled by combining 5 ml of each and were then induced with 4% (final concentration) galactose for three hours. GST-fusion proteins were isolated on glutathione-Sepharose beads as previously described [58]. Isolated proteins conjugated to beads were dried and kept at 4°C no longer than overnight. The biochemical screen followed an iterative process with the first-round pools comprised of eight fusion proteins followed by fractionation of positive pools by halves until single positives were identified.

Expression of full-length *BUD6* and associated fragments was in *E. coli* strain BL21, which was induced with 0.4 mM IPTG for three hours. Fusion proteins were obtained as described [59].

### Protein Kinase Assays

The biochemical screen was designed to screen roughly 10% of the yeast proteome. We reasoned that a substrate responsible for the shared essential function of Ste20p and Cla4p might itself be essential. We thus created a library of 539 essential proteins based on their GO functional and localization annotations [60], reasoning that these would likely still exhibit biochemical diversity characteristic of the protein population as a whole. GO terms used for the selection included broad categories including signal transduction, protein translation or degradation, cell cycle progression, among others.

Kinase assays were as described [33]. Dried beads with GST-fusion proteins bound were resuspended in kinase buffer supplemented with 2 µM ATP and 1 µl [γ-^32^P]-ATP (4,500 Ci/mmol, 10 Ci/µl) and were split in two aliquots, one of which received 1 µl of recombinant GST-Ste20p and the other received an equal volume of protein storage buffer. Reaction mixtures were incubated for 30 minutes and then boiled for 5 minutes after the addition of Laemmli buffer. Samples were separated by SDS-PAGE, dried, and visualized by autoradiography.

### Mass Spectrometry

HPLC grade water and acetonitrile were purchased from Fisher Scientific (Whitby, ON, Canada). Formic acid (FA) and ammonium bicarbonate were obtained from EM Science (Mississauga, ON, Canada). Fused silica capillaries were purchased from Polymicro Technologies (Phoenix, AZ). Jupiter C_18_, 5 µm particle material was from Phenomenex (Torrance, CA).

All LC-MS analyses were performed using a Nano-Acquity Q-TOF Premier (Waters, Millford, MA) with a home-made C_18_ pre-column (5 mm×300 µm i.d. Jupiter 3 µm, C_18_) and an analytical column (10 cm×150 µm i.d., Jupiter 3 µm C_18_). Sample injection was 10 µL, and tryptic digests were first loaded on the pre-column at a flow rate of 4 mL/min and subsequently eluted onto the analytical column using a gradient from 10% to 60% aqueous acetonitrile (0.2% formic acid) over 56 minutes.

### Identification of Predictive Motifs

*S. cerevisiae* protein sequences were obtained from the *Saccharomyces* Genome Database (SGD) in August 2008 [46]. The sequence of each of the 19 known substrates (14 from the *in vitro* screen and five from the literature forming the positive learning set) was scanned with a six-amino acid sliding window to identify sequence fragments where at least three of the residues are predicted to be exposed according to ACCpro 4.0 [44]. The identified fragments (with overlapping fragments merged into single fragments) were then used as input to the Teiresias algorithm [43] in order to identify motifs characterized with regular expressions. The algorithm parameters were set so that identified motifs must contain at least three literal (i.e. non-wildcard) residues and that any three consecutive literals span at most six amino acids.

Each motif *m* was evaluated with respect to the positive and negative (non-substrates of the *in vitro* screen) learning sets by computing its selectivity ratio (*s_m_*) = (Σ*_j_w_j,m_*/*N_pos_*)/( Σ*_k_w_k,m_*/*N_neg_*) where *N_pos_* = 19 is the size of the positive set, *N_neg_* = 525 is the size of the negative set, *w_j,m_* is the total weight of motif *m* for substrate *j* for *j* = 1..*N_pos_* and *w_k,m_* is the total weight of motif *m* for non-substrate *k* for *k* = 1..*N_neg_*. The total weights were computed as shown in Figure 1. The multiple sequence alignments were constructed with ClustalW2 [61] together with *Saccharomyces* sequences and orthology mappings obtained elsewhere [46], [62]. An alignment may include two sequences for proteins in *S. bayanus* and/or *S. mikatae* since for each of these species there were two different research groups that generated sequences. In such an alignment, the conservation score considers whether the motif occurrence was conserved in *any* of the sequences for a given organism and therefore does not double-count. The weights of overlapping motif occurrences were adjusted so that the overlapping region contributes to the weight of only one of the motif occurrence (Figure 1).

### The Predictor of Ste20p Substrates and Its Cross-Validation

The predictor is implemented as a naïve Bayes classifier, and thus computes the posterior probability (i.e. prediction score) that a given sequence *t* encodes a Ste20p substrate as follows:
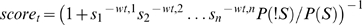
(1)where


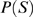
 = 14/539 is the substrate identification rate of the *in vitro* screen (i.e. the prior probability),


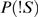
 = 1 - 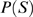 is the non-substrate identification rate of the *in vitro* screen,


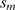
 represents the selectivity ratio for motif 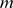,


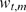
 represents the total weight of motif 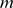 in sequence 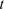, and


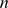
 = 23 is the number of motifs used by the predictor (each with 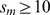).

The *w_t,m_* values used in (1) are computed as in Figure 1 except that conservation is not considered and consequently, *w_t,m_,_i_* = *u_t,m_,_i_* for the *i*th occurrence of motif *m* in sequence *t*. Not considering conservation during prediction allows for added flexibility. For example, it may not be possible to map the sequence of a synthetic peptide to a region of the genome that can be assessed for conservation across species. However, the peptide can still be assessed for the likelihood that it is a Ste20p substrate based on the presence/absence of predictive motifs in its sequence.

Rather than using standard leave-one-out cross-validation where in each iteration, a different (positive or negative) learning example is not used to generate the predictor, we only left out positive examples. A random selection of 100 proteins from the negative set (∼20%) was set aside for testing the predictors resulting from the different iterations. We cross-validated in this way due to the computationally intensive process of deriving selectivity ratios for the thousands of motifs discovered by Teiresias during each iteration.

We experimented with different parameter values and used the overall performance, estimated with the area under the receiver-operator-characteristic curve (AUC), of the resulting predictors to guide the selection of optimal parameter values for the final predictor. For example, the selectivity ratio threshold controls the number of motifs that are used by the predictor, since the smaller the threshold the more motifs that will have selectivity ratios above the threshold, and all motifs that pass the threshold are used. While using more motifs increases the possibility of false positives (i.e. a reduction in specificity), since it becomes more likely for any sequence to contain an occurrence of any predictive motif by chance, the sensitivity of the predictor improves (data not shown). For different parameter values, we estimated the AUC of the resulting predictors using the variation of leave-one-out cross validation described above. The parameter values that result in larger AUCs are better. The optimal parameter values used to build the final predictor are described above.

To integrate the amino acid preferences at the phosphorylation sites of Ste20p-related kinases, we obtained the PSSMs for Pak1, Pak2 and Pak4 [13]. The PSSMs were modified to reflect the background frequencies of amino acids in the *S. cerevisiae* proteome (computed from our collection of protein sequences). Specifically, each PSSM is a 20x10 matrix with entries defined as *x_ij_* = *log*(*p_ij_*/*b_i_*) where *p_ij_* is the probability of observing amino acid *i* at position *j* (in a 10-residue subsequence with the putative phosphorylated residue in the centre), and *b_i_* is the background frequency of amino acid *i*. For any given serine or threonine (S/T) in a sequence *t*, the PSSM score is defined as the sum of the *x_ij_* values corresponding to the observed amino acids flanking the S/T. We define an S/T with a PSSM score greater than or equal to a selected threshold as a likely phosphorylation site of the corresponding kinase.

The selectivity ratio for PSSM *m* with a given score threshold is defined as *s_m_* = (Σ*_j_w_j,m_*/*N_pos_*)/(Σ*_k_w_k,m_*/*N_neg_*), analogous to the selectivity ratio for a regular-expression-based motif. However, here the total weight of PSSM *m* in a sequence *t* is defined as *w_t,m_* = Σ*_i_c_t,m,i_* where *c_t,m,i_* represents the conservation score of the *i*th likely phosphorylation site in *t* according to *m*, and is defined as the fraction of *Saccharomyces* orthologues that also have a likely phosphorylation site according to *m* at the position aligned to the likely site in *S. cerevisiae*. For each PSSM, we computed selectivity ratios using a range of score thresholds and selected the threshold that produced the largest selectivity ratio. The selected threshold for a PSSM *m* and the corresponding *s_m_* are used to integrate *m* into the naïve Bayes classifier (1). As with a regular-expression-based motif, the *w_t,m_* values used in (1) are simplified; here, *w_t,m_* is equal to the number of likely phosphorylation sites in sequence *t* according to PSSM *m* (with the selected threshold).

### Gene Ontology (GO) Analysis

GO slim annotations from all three ontologies were obtained from SGD in August 2008 [46]. The significance of the over-representation of GO category gene sets amongst the predicted substrates was computed using the hypergeometric test in the context of all annotated protein-coding genes. For each ontology, Benjamini and Hochberg multiple-test correction [63] was performed across all categories exhibiting a non-zero overlap with the predicted substrates to obtain adjusted *P* values [63].

### Genetic and Physical Interaction Network Analysis

All *S. cerevisiae* genetic and physical interactions were obtained from BIOGRID v2.0.40 [64]. The networks were reduced to protein-coding genes and self-interactions were removed. To avoid bias and redundancy, Ste20p-substrate interactions from [8] were omitted from the physical network. The neighborhood of a gene/protein is defined as the set of genes/proteins that interact with it in the network. The significance of the overlap between a neighborhood and the set of predicted substrates was computed using the hypergeometric test in the context of all protein-coding genes. For each network, Benjamini and Hochberg multiple-test correction [63] was performed across all neighborhoods exhibiting a non-zero overlap with the predicted substrates to obtain adjusted *P* values.

If a gene/protein has been investigated in multiple interaction studies, it is likely to have more identified interactions compared to a less-studied gene/protein. Consequently, the interaction neighborhood of a frequently studied gene/protein is more likely to significantly overlap with the set of predicted substrates. To correct for this artifact of frequent study, we counted the number of times a gene/protein was used as a bait in interaction screens (*b*). We then considered a linear model that uses *b* to predict the multiple-test corrected *P* value for overlap. The model was trained using data for genes/proteins whose neighborhoods exhibit a non-zero overlap with the set of predicted substrates. The residuals of the model were taken as *P* values adjusted for frequent study (with negative residuals forced to zero).

The one-sided Mann-Whitney test was used to determine whether *STE20* interactor neighborhoods tend to have lower *P* values compared to the neighborhoods of other genes in the network. The same statistical test was performed to determine whether the negative set proteins that are predicted as Ste20p substrates tend to be in more *STE20* GINs compared to all negative set proteins (see Figure S4 and Text S1). We also used GIN and PIN analyses to investigate known false negatives of the predictor (see Table S7 and Text S1).

For Figures 4 and 5, we focused on predicted substrates that are present in at least 1 *STE20* interactor GIN or PIN, respectively. The overlap profiles were clustered using the Ward agglomerative method and the binary distance metric (see hclust documentation in the R statistical computing framework [65]). The significance of branch points in the resulting dendrograms was measured using multiscale bootstrap resampling (see the documentation for the pvclust R package [65]). The Approximately Unbiased (AU) score for a branch point is the percentage of resamples in which the branch point occurs so that larger percentages represent more significant branching.

## Supporting Information

Figure S1 Receiver-Operating Characteristic (ROC) curves of Ste20p substrate predictors. The ROC curves were estimated with a modified version of leave-one-out cross-validation (see Materials and Methods). All predictors are naïve Bayes classifiers that integrate the motifs identified in this study and/or position-specific scoring matrices (PSSMs) that specify the amino acid preferences at the phosphorylation sites of specific Ste20p-related kinases. In the key, “all PAK PSSMs” refers to the Pak1, Pak2 and Pak4 PSSMs. The estimated true and false positive rates of the predictor that only integrates our motifs, used with a threshold of 0.9, are indicated by the dotted red line.
(0.12 MB TIF)

Figure S2 The statistical significance of clusters based on the Genetic Interaction Neighborhood (GIN) analysis shown in Figure 4. Each branch point is labeled with an Approximately Unbiased (AU) score (see Materials and Methods) such that a score ≥95 corresponds to a *P* value ≤0.05 indicating the significance of the cluster. (A) Dendrogram of *STE20* genetic interactors clustered by the overlap of their respective GINs with the set of predicted substrates. The box highlights a cluster containing genes associated with cell-cycle progression and polarized growth. (B) Dendrogram of predicted substrates clustered by their overlap with *STE20*-linked GINs.
(0.78 MB TIF)

Figure S3 The statistical significance of clusters based on the Physical Interaction Neighborhood (PIN) analysis shown in Figure 5. Each branch point is labeled with an Approximately Unbiased (AU) score (see Materials and Methods) such that a score ≥95 corresponds to a *P* value ≤0.05 indicating the significance of the cluster. (A) Dendrogram of Ste20p physical interactors clustered by the overlap of their respective PINs with the set of predicted substrates. (B) Dendrogram of predicted substrates clustered by their overlap with Ste20p-linked PINs. The box highlights a cluster of proteins involved with polarity.
(0.39 MB TIF)

Figure S4 *STE20* genetic neighborhood analysis suggests that several predicted substrates in the negative set may represent false negatives of the biochemical screen. There are 34 negative set proteins that are predicted as substrates and some are present in the neighborhoods of *STE20* genetic interactors (i.e., a table cell is red if the predicted substrate of the column is present in the genetic neighborhood of the gene of the row, white otherwise). In general, the negative proteins predicted as substrates are present in more neighborhoods compared to all proteins in the negative set (*P* ≅ 3.17×10^−5^, Mann-Whitney test). See Figure 3A for an illustration of an interaction neighborhood.
(0.44 MB TIF)

Table S1 The substrate prediction scores of all yeast proteins.
(0.73 MB XLS)

Table S2 GO slim Cellular Component analysis of predicted Ste20p substrates (score ≥0.9).
(0.03 MB DOC)

Table S3 GO slim Biological Process analysis of predicted Ste20p substrates (score ≥0.9).
(0.03 MB DOC)

Table S4 GO slim Molecular Function analysis of predicted Ste20p substrates (score ≥0.9).
(0.03 MB DOC)

Table S5 Predicted substrates (score ≥0.9) in the neighborhoods of *STE20* genetic interactors.
(0.06 MB DOC)

Table S6 Predicted substrates (score ≥0.9) in the neighborhoods of Ste20p physical interactors.
(0.05 MB DOC)

Table S7 The number of *STE20*-linked Genetic Interaction Neighborhoods (GINs) and Ste20p-linked Physical Interaction Neighborhoods (PINs) in which each known Ste20p substrate (from the positive learning set) appears.
(0.04 MB DOC)

Text S1 Analysis of the predictor that integrates the amino acid preferences at the phosphorylation sites of specific Ste20p-related kinases. GIN and PIN analyses to investigate the known false negatives and false positives generated by the predictor.
(0.03 MB DOC)
