# Supplementary figures and images for: A Biochemical Genomics Screen for Substrates of Ste20p Kinase Enables the In Silico Prediction of Novel Substrates
Source: PLoS One. 2009 Dec 16;4(12):e8279. doi: 10.1371/journal.pone.0008279 (PMC2791418; doi:10.1371/journal.pone.0008279)

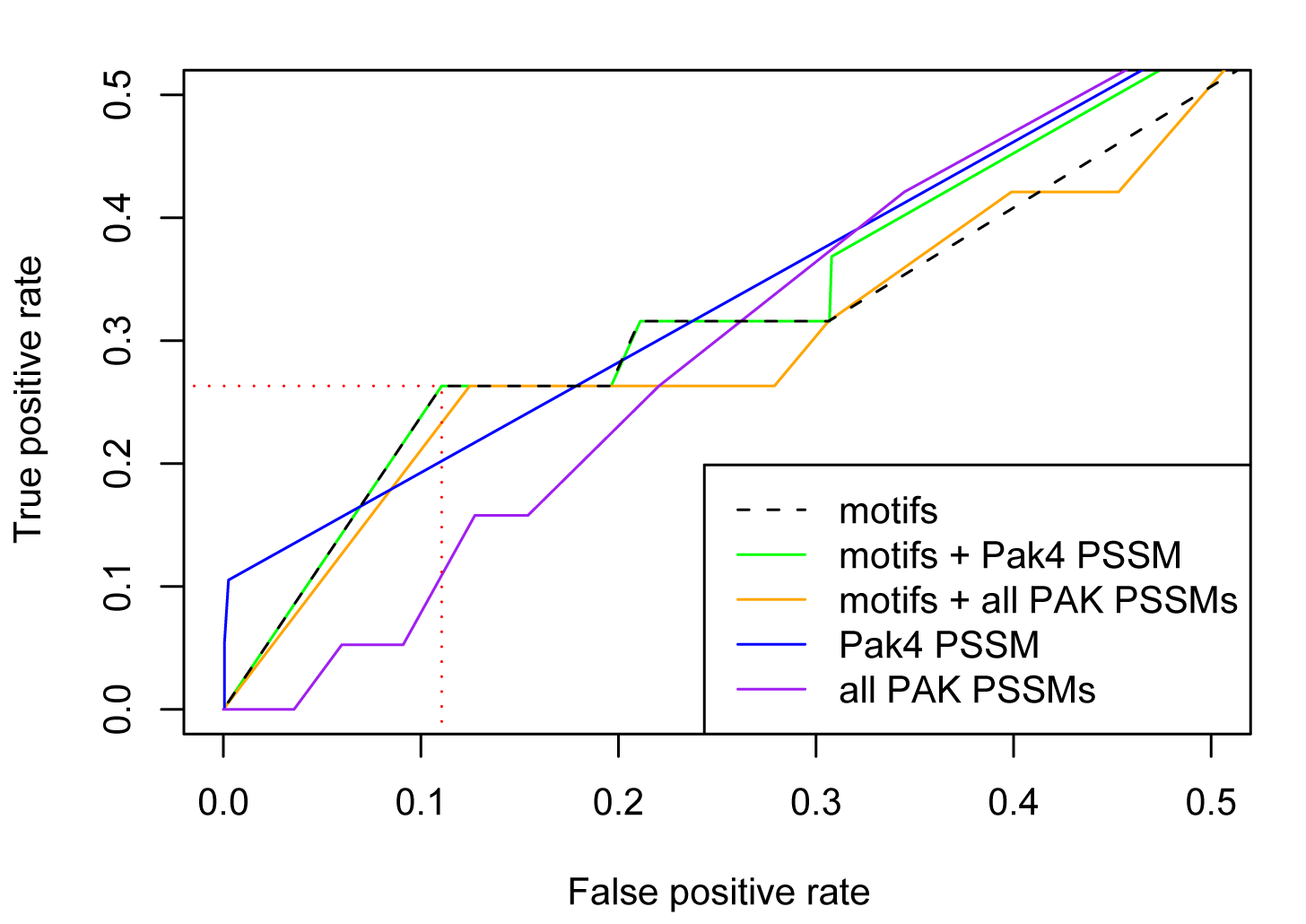

Supplement: Figure S1 — Receiver-Operating Characteristic (ROC) curves of Ste20p substrate predictors. The ROC curves were estimated with a modified version of leave-one-out cross-validation (see Materials and Methods). All predictors are naïve Bayes classifiers that integrate the motifs identified in this study and/or position-specific scoring matrices (PSSMs) that specify the amino acid preferences at the phosphorylation sites of specific Ste20p-related kinases. In the key, “all PAK PSSMs” refers to the Pak1, Pak2 and Pak4 PSSMs. The estimated true and false positive rates of the predictor that only integrates our motifs, used with a threshold of 0.9, are indicated by the dotted red line. (0.12 MB TIF) [file pone.0008279.s001.tif]

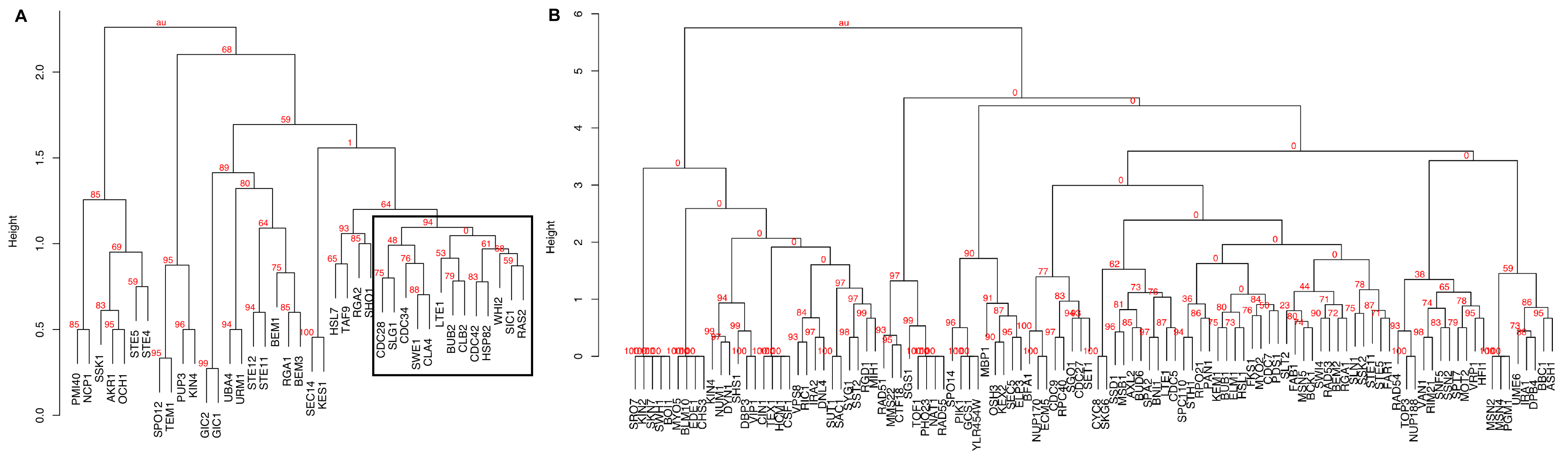

Supplement: Figure S2 — The statistical significance of clusters based on the Genetic Interaction Neighborhood (GIN) analysis shown in Figure 4. Each branch point is labeled with an Approximately Unbiased (AU) score (see Materials and Methods) such that a score ≥95 corresponds to a P value ≤0.05 indicating the significance of the cluster. (A) Dendrogram of STE20 genetic interactors clustered by the overlap of their respective GINs with the set of predicted substrates. The box highlights a cluster containing genes associated with cell-cycle progression and polarized growth. (B) Dendrogram of predicted substrates clustered by their overlap with STE20-linked GINs. (0.78 MB TIF) [file pone.0008279.s002.tif]

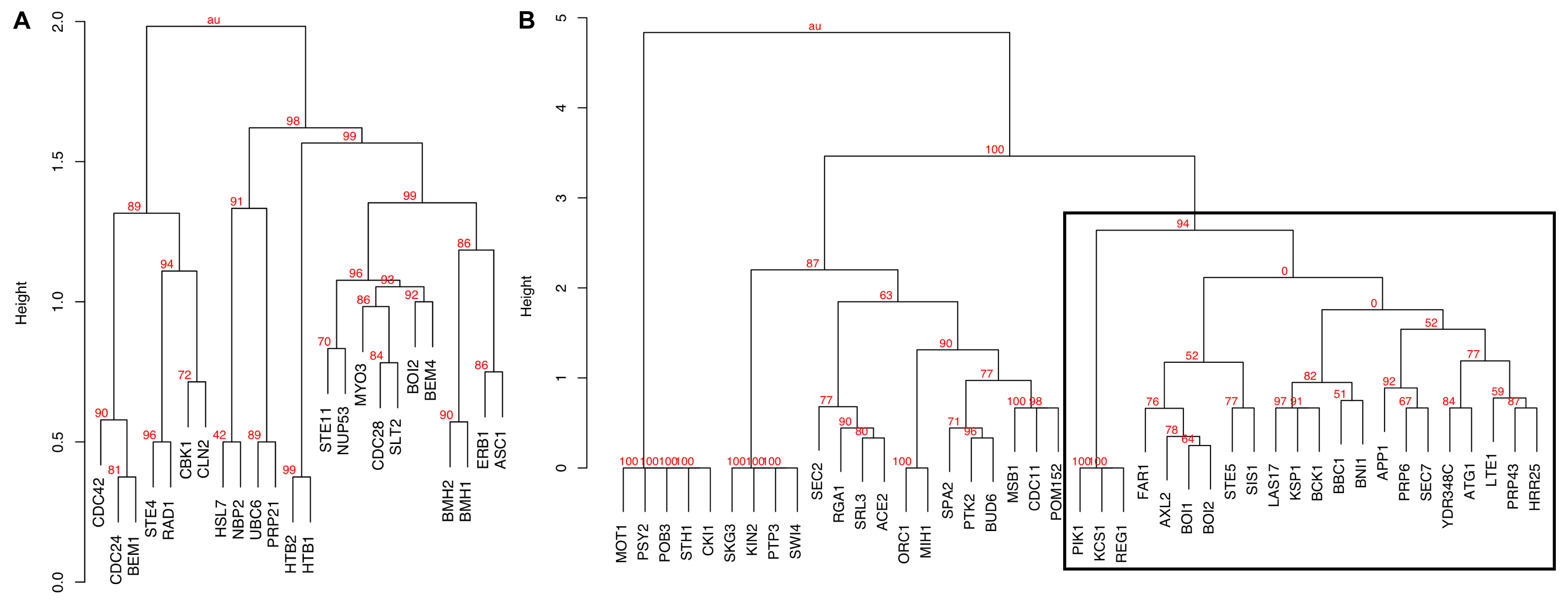

Supplement: Figure S3 — The statistical significance of clusters based on the Physical Interaction Neighborhood (PIN) analysis shown in Figure 5. Each branch point is labeled with an Approximately Unbiased (AU) score (see Materials and Methods) such that a score ≥95 corresponds to a P value ≤0.05 indicating the significance of the cluster. (A) Dendrogram of Ste20p physical interactors clustered by the overlap of their respective PINs with the set of predicted substrates. (B) Dendrogram of predicted substrates clustered by their overlap with Ste20p-linked PINs. The box highlights a cluster of proteins involved with polarity. (0.39 MB TIF) [file pone.0008279.s003.tif]

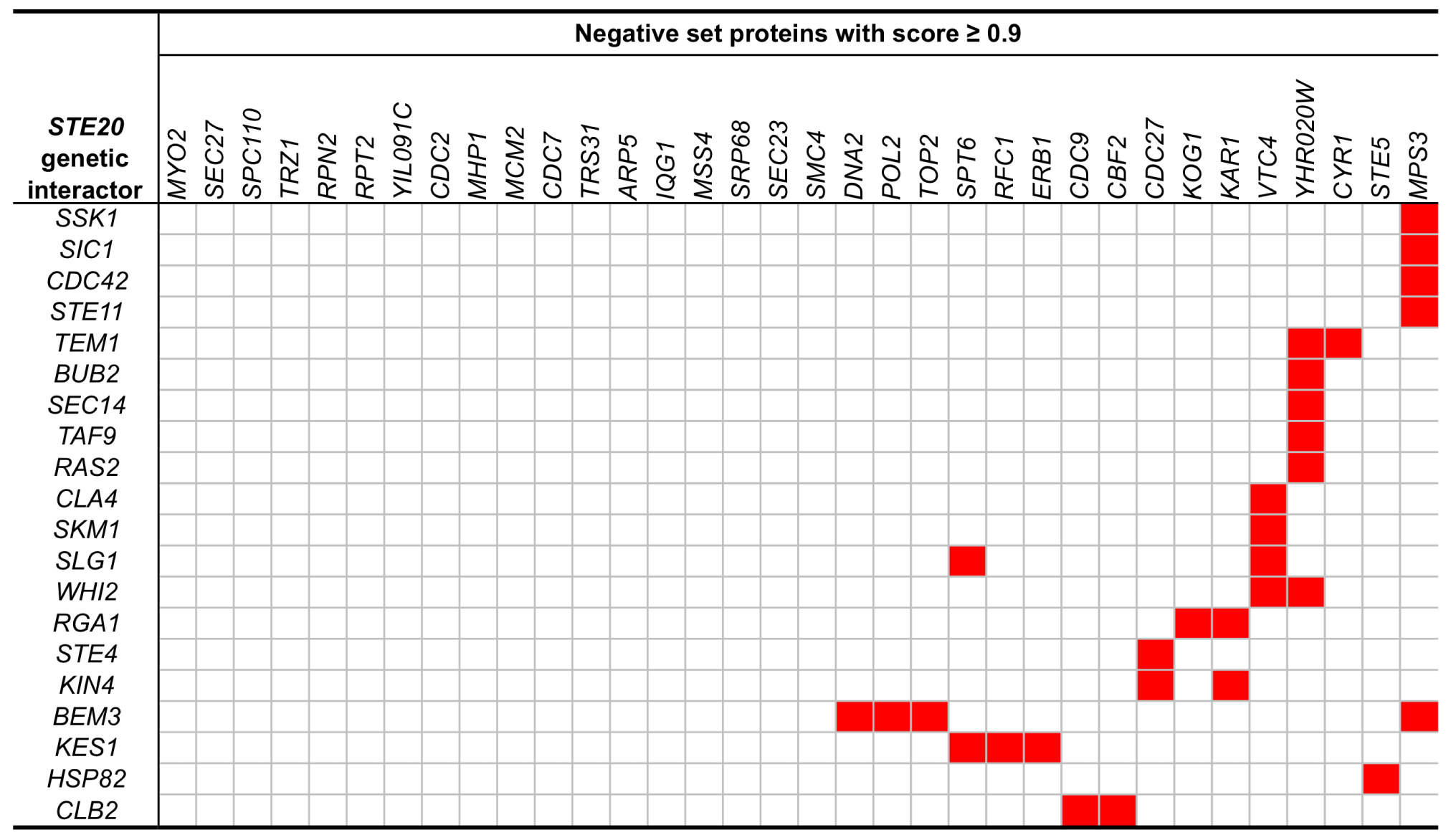

Supplement: Figure S4 — STE20 genetic neighborhood analysis suggests that several predicted substrates in the negative set may represent false negatives of the biochemical screen. There are 34 negative set proteins that are predicted as substrates and some are present in the neighborhoods of STE20 genetic interactors (i.e., a table cell is red if the predicted substrate of the column is present in the genetic neighborhood of the gene of the row, white otherwise). In general, the negative proteins predicted as substrates are present in more neighborhoods compared to all proteins in the negative set (P ≅ 3.17×10^−5^, Mann-Whitney test). See Figure 3A for an illustration of an interaction neighborhood. (0.44 MB TIF) [file pone.0008279.s004.tif]
