# Supplementary material for: A Biochemical Genomics Screen for Substrates of Ste20p Kinase Enables the In Silico Prediction of Novel Substrates
Source: PLoS One. 2009 Dec 16;4(12):e8279. doi: 10.1371/journal.pone.0008279 (PMC2791418; doi:10.1371/journal.pone.0008279)
Supplement: Table S2 — GO slim Cellular Component analysis of predicted Ste20p substrates (score ≥0.9). (0.03 MB DOC) [file pone.0008279.s006.doc]

**Table S2.** GO slim Cellular Component analysis of predicted Ste20p substrates (score ≥ 0.9).

| GO Slim Term | GO Slim Term Size | Overlap Size | *P* value | Adjusted *P* value |
| --- | --- | --- | --- | --- |
| cellular bud | 160 | 47 | 3.71E-11 | 9.28E-10 |
| site of polarized growth | 162 | 45 | 7.47E-10 | 9.34E-09 |
| cell cortex | 105 | 33 | 5.04E-09 | 4.20E-08 |
| plasma membrane | 265 | 53 | 3.68E-06 | 2.30E-05 |
| mitochondrion | 1051 | 155 | 5.18E-06 | 2.59E-05 |
| cytoskeleton | 203 | 43 | 6.92E-06 | 2.88E-05 |
| cytoplasm | 2834 | 345 | 0.000476 | 0.001699 |
| vacuole | 202 | 34 | 0.004684 | 0.014638 |
| nucleus | 1794 | 221 | 0.005773 | 0.016035 |
| Golgi apparatus | 215 | 34 | 0.012190 | 0.030476 |
| other | 384 | 54 | 0.020105 | 0.045693 |
| cytoplasmic membrane-bounded vesicle | 103 | 18 | 0.024088 | 0.046322 |
| chromosome | 249 | 37 | 0.023283 | 0.046322 |
| membrane | 911 | 115 | 0.026463 | 0.047256 |
