# Supplementary material for: A Biochemical Genomics Screen for Substrates of Ste20p Kinase Enables the In Silico Prediction of Novel Substrates
Source: PLoS One. 2009 Dec 16;4(12):e8279. doi: 10.1371/journal.pone.0008279 (PMC2791418; doi:10.1371/journal.pone.0008279)
Supplement: Table S3 — GO slim Biological Process analysis of predicted Ste20p substrates (score ≥0.9). (0.03 MB DOC) [file pone.0008279.s007.doc]

**Table S3.** GO slim Biological Process analysis of predicted Ste20p substrates (score ≥ 0.9).

| GO Slim Term | GO Slim Term Size | Overlap Size | *P* value | Adjusted *P* value |
| --- | --- | --- | --- | --- |
| transcription | 585 | 105 | 0.00019 | 0.00721 |
| anatomical structure morphogenesis | 156 | 35 | 0.00073 | 0.01379 |
| cell budding | 84 | 21 | 0.00207 | 0.02622 |
| carbohydrate metabolic process | 189 | 38 | 0.00368 | 0.03498 |
| vesicle-mediated transport | 340 | 61 | 0.00477 | 0.03626 |
| cytokinesis | 116 | 25 | 0.00698 | 0.04424 |
