# Supplementary material for: A Biochemical Genomics Screen for Substrates of Ste20p Kinase Enables the In Silico Prediction of Novel Substrates
Source: PLoS One. 2009 Dec 16;4(12):e8279. doi: 10.1371/journal.pone.0008279 (PMC2791418; doi:10.1371/journal.pone.0008279)
Supplement: Table S4 — GO slim Molecular Function analysis of predicted Ste20p substrates (score ≥0.9). (0.03 MB DOC) [file pone.0008279.s008.doc]

**Table S4.** GO slim Molecular Function analysis of predicted Ste20p substrates (score ≥ 0.9).

| GO Slim Term | GO Slim Term Size | Overlap Size | *P* value | Adjusted *P* value |
| --- | --- | --- | --- | --- |
| protein kinase activity | 129 | 36 | 0.00001 | 0.00027 |
| lipid binding | 53 | 19 | 0.00004 | 0.00042 |
| hydrolase activity | 725 | 131 | 0.00013 | 0.00099 |
