# Supplementary material for: A Biochemical Genomics Screen for Substrates of Ste20p Kinase Enables the In Silico Prediction of Novel Substrates
Source: PLoS One. 2009 Dec 16;4(12):e8279. doi: 10.1371/journal.pone.0008279 (PMC2791418; doi:10.1371/journal.pone.0008279)
Supplement: Table S5 — Predicted substrates (score ≥0.9) in the neighborhoods of STE20 genetic interactors. (0.06 MB DOC) [file pone.0008279.s009.doc]

**Table S5.** Predicted substrates (score ≥ 0.9) in the neighborhoods of *STE20* genetic interactors.

| *STE20* Genetic Interactor | Neighborhood Size | Overlap Size | *P* value | Adjusted *P* value |
| --- | --- | --- | --- | --- |
| *CDC42* | 84 | 26 | 8.39E-07 | 0.001982 |
| *SWE1* | 45 | 16 | 1.58E-05 | 0.006404 |
| *GIC2* | 24 | 10 | 0.000138 | 0.031707 |
| *BUB2* | 86 | 21 | 0.000423 | 0.042764 |
| *HSP82* | 1729 | 233 | 0.000507 | 0.042764 |
| *RAS2* | 136 | 28 | 0.001025 | 0.060549 |
| *CLA4* | 197 | 36 | 0.002091 | 0.080261 |
| *GIC1* | 28 | 9 | 0.002646 | 0.090666 |
| *SLG1* | 36 | 10 | 0.005113 | 0.118427 |
| *TAF9* | 141 | 25 | 0.013734 | 0.173212 |
| *STE4* | 30 | 8 | 0.015272 | 0.178672 |
| *KES1* | 25 | 7 | 0.017428 | 0.195614 |
| *BEM1* | 50 | 11 | 0.020991 | 0.206127 |
| *CDC28* | 61 | 12 | 0.037011 | 0.243204 |
| *STE12* | 44 | 9 | 0.053209 | 0.294332 |
| *WHI2* | 72 | 13 | 0.056568 | 0.305749 |
| *PMI40* | 4 | 2 | 0.065104 | 0.311286 |
| *BEM3* | 14 | 4 | 0.063669 | 0.311286 |
| *SEC14* | 61 | 11 | 0.076094 | 0.343404 |
| *HSL7* | 11 | 3 | 0.118118 | 0.356772 |
| *CDC34* | 45 | 8 | 0.127147 | 0.379673 |
| *SIC1* | 150 | 21 | 0.171830 | 0.442463 |
| *RGA1* | 13 | 3 | 0.173463 | 0.442463 |
| *SHO1* | 7 | 2 | 0.181741 | 0.444381 |
| *AKR1* | 8 | 2 | 0.225106 | 0.460346 |
| *KIN4* | 8 | 2 | 0.225106 | 0.460346 |
| *STE5* | 16 | 3 | 0.266171 | 0.511751 |
| *PUP3* | 3 | 1 | 0.301282 | 0.514553 |
| *CLB2* | 79 | 11 | 0.272907 | 0.514553 |
| *LTE1* | 269 | 33 | 0.325320 | 0.543426 |
| *RGA2* | 4 | 1 | 0.380007 | 0.554402 |
| *SSK1* | 11 | 2 | 0.356320 | 0.554402 |
| *STE11* | 45 | 6 | 0.396462 | 0.568879 |
| *SPO12* | 21 | 3 | 0.426868 | 0.603028 |
| *TEM1* | 21 | 3 | 0.426868 | 0.603028 |
| *NCP1* | 5 | 1 | 0.449874 | 0.603408 |
| *UBA4* | 13 | 2 | 0.439535 | 0.603408 |
| *OCH1* | 8 | 1 | 0.615726 | 0.706234 |
| *URM1* | 12 | 1 | 0.761898 | 0.805036 |
| *MSB2* | 7 | 0 | 1.000000 | N/A* |
| *STE3* | 5 | 0 | 1.000000 | N/A* |
| *SKM1* | 2 | 0 | 1.000000 | N/A* |

See Figure 3A for an illustration of an interaction neighborhood.

*Adjusted *P* values were not computed for genetic interactors with neighborhoods that do not overlap with the predicted substrates.
