# Supplementary material for: A Biochemical Genomics Screen for Substrates of Ste20p Kinase Enables the In Silico Prediction of Novel Substrates
Source: PLoS One. 2009 Dec 16;4(12):e8279. doi: 10.1371/journal.pone.0008279 (PMC2791418; doi:10.1371/journal.pone.0008279)
Supplement: Table S6 — Predicted substrates (score ≥0.9) in the neighborhoods of Ste20p physical interactors. (0.05 MB DOC) [file pone.0008279.s010.doc]

**Table S6.** Predicted substrates (score ≥ 0.9) in the neighborhoods of Ste20p physical interactors.

| Ste20p Physical Interactor | Neighborhood Size | Overlap Size | *P* value | Adjusted *P* value |
| --- | --- | --- | --- | --- |
| Cdc28p | 241 | 67 | 4.81E-13 | 1.44E-09 |
| Bem1p | 26 | 10 | 0.000304 | 0.053593 |
| Htb2p | 81 | 18 | 0.003308 | 0.153509 |
| Myo3p | 30 | 9 | 0.004460 | 0.189745 |
| Bmh1p | 51 | 12 | 0.009550 | 0.258371 |
| Cdc42p | 29 | 8 | 0.012388 | 0.258371 |
| Slt2p | 48 | 11 | 0.015618 | 0.290825 |
| Cbk1p | 25 | 7 | 0.017428 | 0.311015 |
| Asc1p | 50 | 11 | 0.020991 | 0.328345 |
| Cdc24p | 23 | 6 | 0.037730 | 0.405232 |
| Cbr1p | 18 | 5 | 0.044005 | 0.448729 |
| Hsl7p | 8 | 3 | 0.051646 | 0.463581 |
| Ste11p | 25 | 6 | 0.054610 | 0.465113 |
| Ubc6p | 14 | 3 | 0.203407 | 0.630751 |
| Bmh2p | 67 | 10 | 0.217466 | 0.641066 |
| Prp21p | 42 | 6 | 0.333462 | 0.702544 |
| Nup53p | 26 | 4 | 0.335328 | 0.702638 |
| Ste4p | 26 | 4 | 0.335328 | 0.702638 |
| Nbp2p | 4 | 1 | 0.380007 | 0.705863 |
| Htb1p | 67 | 9 | 0.339866 | 0.705863 |
| Ncp1p | 13 | 2 | 0.439535 | 0.731411 |
| Cln2p | 46 | 6 | 0.417478 | 0.731411 |
| Bem4p | 16 | 2 | 0.552262 | 0.785380 |
| Boi2p | 10 | 1 | 0.697505 | 0.846607 |
| Rad1p | 31 | 3 | 0.694673 | 0.846607 |
| Erb1p | 84 | 8 | 0.744227 | 0.874634 |
| Boi1p | 13 | 1 | 0.788761 | 0.889489 |
| Erg4p | 2 | 0 | 1.000000 | N/A* |
| Bud8p | 4 | 0 | 1.000000 | N/A* |

See Figure 3A for an illustration of an interaction neighborhood.

*Adjusted *P* values were not computed for physical interactors with neighborhoods that do not overlap with the predicted substrates.
