# Supplementary material for: A Biochemical Genomics Screen for Substrates of Ste20p Kinase Enables the In Silico Prediction of Novel Substrates
Source: PLoS One. 2009 Dec 16;4(12):e8279. doi: 10.1371/journal.pone.0008279 (PMC2791418; doi:10.1371/journal.pone.0008279)
Supplement: Table S7 — The number of STE20-linked Genetic Interaction Neighborhoods (GINs) and Ste20p-linked Physical Interaction Neighborhoods (PINs) in which each known Ste20p substrate (from the positive learning set) appears. (0.04 MB DOC) [file pone.0008279.s011.doc]

**Table S7.** The number of *STE20*-linked Genetic Interaction Neighborhoods (GINs) and Ste20p-linked Physical Interaction Neighborhoods (PINs) in which each known Ste20p substrate (from the positive learning set) appears.

| Gene | Predictor Score | # Overlapping *STE20*-linked GINs (out of 42) | # Overlapping Ste20p-linked PINs (out of 29) |
| --- | --- | --- | --- |
| *ALY2* | 1.00 | 1 | 1 |
| *BMS1* | 1.00 | 0 | 0 |
| *CDC3* | 1.00 | 0 | 1 |
| *MYO3* | 1.00 | 1 | 1 |
| *MYO5* | 1.00 | 2 | 1 |
| *RAD53* | 1.00 | 3 | 1 |
| *RPT5* | 1.00 | 0 | 0 |
| *SGV1* | 1.00 | 1 | 1 |
| *SPB1* | 1.00 | 0 | 0 |
| *STE11* | 1.00 | 12 | 1 |
| *UTP7* | 1.00 | 0 | 1 |
| *COG4* | 0.99 | 0 | 0 |
| *PCM1* | 0.99 | 1 | 0 |
| *CDC10* | 0.86 | 2 | 0 |
| *RSC6* | 0.28 | 3 | 0 |
| *HTB2* | 0.03 | 0 | 1 |
| *RSC8* | 0.03 | 1 | 0 |
| *SPT16* | 0.03 | 0 | 3 |
| *UTP5* | 0.03 | 0 | 0 |

See Figure 3A for an illustration of an interaction neighborhood.
