# Supplementary material for: A Biochemical Genomics Screen for Substrates of Ste20p Kinase Enables the In Silico Prediction of Novel Substrates
Source: PLoS One. 2009 Dec 16;4(12):e8279. doi: 10.1371/journal.pone.0008279 (PMC2791418; doi:10.1371/journal.pone.0008279)
Supplement: Text S1 — Analysis of the predictor that integrates the amino acid preferences at the phosphorylation sites of specific Ste20p-related kinases. GIN and PIN analyses to investigate the known false negatives and false positives generated by the predictor. (0.03 MB DOC) [file pone.0008279.s012.doc]

**Text S1**

Rennefahrt and colleagues determined amino acid preferences at the phosphorylation sites of the human Ste20p-related kinases Pak1 (Ensembl:ENSG00000149269), Pak2 (Ensembl:ENSG00000180370) and Pak4 (Ensembl:ENSG00000130669) [1]. For each kinase, the preferences are encoded in a position-specific scoring matrix (PSSM) that can be used to score each serine and threonine in any given sequence. All serines and threonines with a PSSM score that exceeds a selected threshold are deemed likely phosphorylation sites of the kinase. We computed a selectivity ratio for each PSSM (similar to the selectivity ratios of the motifs identified in this study, see Materials and Methods) such that a PSSM with a selectivity ratio greater than one indicates that likely phosphorylation sites are more prevalent in the positive set than in the negative set. The selectivity ratios for all the PAK PSSMs are less than the ratio threshold (i.e. 10) we used to select regular-expression-based motifs for the predictor (the selectivity ratios for the Pak1, Pak2, and Pak4 PSSMs are 1.66, 2.30 and 7.88, respectively). A classifier that integrates the motifs identified in this study with the PSSM with the largest selectivity ratio (i.e. the Pak4 PSSM) is approximately as accurate as the classifier that only uses our motifs (Figure S1, see Materials and Methods). A naïve Bayes classifier that integrates our motifs with all the PSSMs also has comparable accuracy (Figure S1). Taken together, our results show that integrating the PAK PSSMs does not improve the accuracy of our Ste20p substrate predictor.

We also employed GIN and PIN analyses to investigate the known false negatives and false positives generated by the predictor. Five of the 19 Ste20p substrates identified in our initial screen or from the literature were poorly scored (score < 0.3) by the predictor (Table S7). Four of these are members of the GINs or PINs of Ste20 interactors, suggesting that they retain *in vivo* relevance but do not possess the sequence features used by the predictor to characterize Ste20p substrates. These represent likely false negatives of the predictor. Furthermore, among the set of proteins that were not phosphorylated in our screen, 34 proteins were predicted as Ste20p substrates (Figure S4). These proteins tend to be found surprisingly often in the GINs of *STE20* interactors compared to all the proteins from the negative learning set (*P* ≈ 3.17×10^-5^, Mann-Whitney test). These proteins therefore represent likely false negatives of the *in vitro* screen yet are correctly predicted substrates.

**Reference**

1. Rennefahrt UE, Deacon SW, Parker SA, Devarajan K, Beeser A, et al. (2007) Specificity profiling of Pak kinases allows identification of novel phosphorylation sites. J Biol Chem 282: 15667-15678.
